# Stepped Geomorphology Shaped the Phylogeographic Structure of a Widespread Tree Species (*Toxicodendron vernicifluum*, Anacardiaceae) in East Asia

**DOI:** 10.3389/fpls.2022.920054

**Published:** 2022-06-02

**Authors:** Lu Wang, Yao Li, Shuichi Noshiro, Mitsuo Suzuki, Takahisa Arai, Kazutaka Kobayashi, Lei Xie, Mingyue Zhang, Na He, Yanming Fang, Feilong Zhang

**Affiliations:** ^1^Co-Innovation Center for Sustainable Forestry in Southern China, Key Laboratory of State Forestry and Grassland Administration on Subtropical Forest Biodiversity Conservation, College of Biology and the Environment, Nanjing Forestry University, Nanjing, China; ^2^Center for Obsidian and Lithic Studies, Meiji University, Tokyo, Japan; ^3^Botanical Gardens, Tohoku University, Sendai, Japan; ^4^Xi’an Research Institute of Chinese Lacquer, All China Federation of Supply and Marketing Cooperatives, Xi’an, China

**Keywords:** chloroplast haplotype, dispersal corridor, East Asia, geological isolation, phylogeographic break, refugia, stepped geomorphology, *Toxicodendron vernicifluum*

## Abstract

Species’ phylogeographic patterns reflect the interplay between landscape features, climatic forces, and evolutionary processes. Here, we used two chloroplast DNA (cpDNA) markers (*trnL* and *trnL*-*F*) to explore the role of stepped geomorphology in shaping the phylogeographic structure of *Toxicodendron vernicifluum*, an economically important tree species widely distributed in East Asia. The range-wide pattern of sequence variation was analyzed based on a dataset including 357 individuals from China, together with published sequences of 92 individuals mainly from Japan and South Korea. We identified five chloroplast haplotypes based on seven substitutions across the 717-bp alignment. A clear east-west phylogeographic break was recovered according to the stepped landforms of mainland China. The wild trees of the western clade were found to be geographically restricted to the “middle step”, which is characterized by high mountains and plateaus, while those of the eastern clade were confined to the “low step”, which is mainly made up of hills and plains. The two major clades were estimated to have diverged during the Early Pleistocene, suggesting that the cool glacial climate may have caused the ancestral population to retreat to at least two glacial refugia, leading to allopatric divergence in response to long-term geographic isolation. Migration vector analyses based on the outputs of ecological niche models (ENMs) supported a gradual range expansion since the Last Interglacial. Mountain ranges in western China and the East China Sea land bridge were inferred to be dispersal corridors in the western and eastern distributions of *T. vernicifluum*, respectively. Overall, our study provides solid evidence for the role of stepped geomorphology in shaping the phylogeographic patterns of *T. vernicifluum*. The resulting east-west genetic discontinuities could persist for a long time, and could occur at a much larger scale than previously reported, extending from subtropical (e.g., the Xuefeng Mountain) to warm-temperate China (e.g., the Taihang Mountain).

## Introduction

Species’ phylogeographic patterns contain valuable information regarding the impacts of past climatic and geological events on neutral evolutionary processes such as gene flow and genetic drift (Taberlet et al., 1998; Avise, 2000; Hewitt, 2004; Hickerson et al., 2010; Qiu et al., 2011; Feliner, 2014). One of the most remarkable genetic legacies within such patterns is the occurrence of phylogeographic breaks, where intraspecific gene flow is highly restricted and distinct lineages are geographically separated (Avise, 1992; Soltis et al., 1997, 2006; Schaal et al., 1998; Ye et al., 2017a). These breaks may arise when allopatric populations have experienced long-term isolation across major physiographic barriers to dispersals, such as mountains, rivers, glaciers, and oceans (Soltis et al., 2006; Jaramillo-Correa et al., 2009). However, sometimes obvious physical barriers are absent and climate or habitat barriers play a more important role (Geffen et al., 2004; Bai et al., 2016; Cab-Sulub and Álvarez-Castañeda, 2021). In a given area, co-distributed species may exhibit common phylogeographic breaks because they have a shared biogeographic history (Arbogast and Kenagy, 2001; Soltis et al., 2006), but diverse patterns are more frequently observed reflecting the complex history affected by not only a few barriers (Soltis et al., 2006; Shafer et al., 2010; Fan et al., 2016).

East Asia harbors greater species diversity due to its extreme topographic complexity and physiographical heterogeneity (Qian and Ricklefs, 2000; Yin et al., 2021). The absence of continental glaciation and relatively low climate change velocity contribute to the preservation of high plant endemism (Feng et al., 2016). These factors also shape the geographic distributions of intraspecific genealogical lineages across the landscape, allowing long-term refugial isolation and *in situ* survival of local populations in both subtropical and warm-temperate areas (e.g., Wang et al., 2009, 2015a,b; Sakaguchi et al., 2012; Kou et al., 2016; Li et al., 2019), even in cool-temperate regions (e.g., Hu et al., 2008; Zeng et al., 2015; Zhang et al., 2015; Ye et al., 2017b). Nevertheless, climate cooling since the mid-Miocene, the uplift of the Qinghai-Tibetan Plateau (QTP), the intensification of the East Asian monsoon, and repeated sea-level changes during the Pleistocene were shown to have strongly influenced the evolutionary history of local plants (e.g., Gao et al., 2007; Chen et al., 2012; Luo et al., 2016; Jiang et al., 2021; Li et al., 2022). These contexts determine that East Asian plants display distinct phylogeographic patterns in comparison with those on other continents (Qiu et al., 2011, 2017).

Previous phylogeographic studies have confirmed that both north-south and east-west genetic discontinuities are common patterns for temperate and subtropical plants in East Asia (Qiu et al., 2017; Ye et al., 2017b). A clear north-south phylogeographic split has been reported for widespread tree species such as *Acer mono* (Guo et al., 2014; Liu et al., 2014), *Juglans* spp. (Bai et al., 2016), and *Lindera obtusiloba* (Ye et al., 2017b). This break is closely associated with an east-west orientated arid belt that has existed during the Paleogene and redeveloped during the late Miocene (Guo et al., 2008). The belt was inferred to have acted as a climate barrier that impeded the migration across it and resulted in the late Miocene diversification of Tertiary relict plants (Bai et al., 2016; Ye et al., 2017a). Furthermore, the boundary between the subtropical and tropical regions was found to have shaped the north-south patterns of dominant species in evergreen broadleaved subtropical forests (e.g., *Lindera aggregata*, Ye and Li, 2021).

East-west phylogeographic splits were more commonly observed in East Asian plants (Qiu et al., 2017; Ye et al., 2017a). Previous studies have identified several phylogeographic breaks, coinciding with the East China Sea (ECS; e.g., *Platycrater arguta*, Qi et al., 2014; *Euptelea* spp., Cao et al., 2016) or with the boundary between the Sino-Himalayan and Sino-Japanese Forest subkingdoms (e.g., *Taxus wallichiana*, Gao et al., 2007; *Davidia involucrata*, Luo et al., 2011; and *Sophora davidii*, Fan et al., 2013). More interestingly, a recent study demonstrated that the stepped landforms of mainland China, together with the ECS, play a more important role in shaping the distinct phylogeographic structure of a widespread shrub, *Kerria japonica* (Luo et al., 2021). The geomorphology of China is characterized by three giant “steps”: the high (average ~4,000 m, e.g., the QTP), middle (average ~2,000 m, e.g., the Yunnan-Guizhou Plateau and the Qinling Mountains), and low (average <500 m, e.g., the plains and hills in eastern China) “steps” spanning from the west to the east (Jiang and Wu, 1993; Wan, 2012). The mountain ranges between these three areas may have served as geographic barriers that prevent gene flow and further facilitate population differentiation (Li et al., 2015). Indeed, limited chloroplast haplotype sharing between the middle and low “steps” has been reported for several woody and herbaceous plants in subtropical China (e.g., *Juglans cathayensis* Bai et al., 2014; *Boea clarkeana*, Wang et al., 2018; *Liriodendron chinense*, Yang et al., 2019) as well as a few widespread tree species in East Asia (e.g., *Kalopanax septemlobus*, Sakaguchi et al., 2012).

In this study, we used chloroplast DNA (cpDNA) to explore the role of stepped geomorphology in shaping the phylogeographic pattern of *Toxicodendron vernicifluum* (Stokes) F. A. Barkley, a deciduous and dioecious tree widely distributed in temperate and subtropical areas of East Asia. This species belongs to the family Anacardiaceae and is commonly known as lacquer tree (also called “qishu” in Chinese, “urushi” in Japanese, and “otnamu” in Korean; Hashida et al., 2014; Suzuki et al., 2014; Kim et al., 2015; Wang et al., 2020a). It has been cultivated in East Asian countries (China, Korea, and Japan) for thousands of years, whose toxic sap is traditionally used as a highly durable lacquer to make lacquerware (Noshiro and Suzuki, 2004; Walker et al., 2008; Zhao et al., 2013; Suzuki et al., 2014; Wu et al., 2018; Li et al., 2021). In western China, wild lacquer trees usually grow in mountain forests at an altitude between 800 and 2,800 m. It is mainly distributed in the mountainous areas surrounding the Sichuan Basin (e.g., the Qinling Mountains, the Daba Mountains, the Wuling Mountains, the Dalou Mountains, and the Wumeng Mountains). In eastern China, natural forests of lacquer trees are scattered in hilly areas at an altitude less than 600 m, such as those in Liaoning and Shandong Provinces (Suzuki et al., 2007; Zhang et al., 2007). It is believed that lacquer trees in Japan and Korea are introduced from mainland China (Noshiro and Suzuki, 2004). However, a fossil wood dating back to the incipient Jomon period (~12,600 years ago) suggested the species is likely to be native to Japan (Suzuki et al., 2014).

A previous study has used two cpDNA fragments to examine the phylogenetic relationships between lacquer trees sampled from mainland China and Japan (Suzuki et al., 2014). They found that populations in eastern China and Japan shared a haplotype, while those in western China harbored another haplotype, suggesting that the stepped landforms in China may have shaped the present phylogeographic patterns of *T. vernicifluum*. However, this study only sampled a limited number of lacquer trees from China. Other researchers also used various molecular markers (e.g., amplified fragment length polymorphisms and nuclear microsatellites) to investigate the genetic variation patterns of *T. vernicifluum* at different scales (Wei et al., 2010; Bai et al., 2017; Bui et al., 2017; Vu et al., 2018; Guo et al., 2019; Watanabe et al., 2019), but their conclusions are mainly restricted by small sample size and a narrow sampling range. Here, we used the two cpDNA makers of Suzuki et al. (2014) to sequence the samples from 39 sites that encompass the entire natural range of *T. vernicifluum* in China. The obtained dataset was combined with that of Suzuki et al. (2014) to investigate the phylogeographic structure of *T. vernicifluum* throughout East Asia. We aimed to: (1) examine range-wide patterns of cpDNA variation and identify potential phylogeographic breaks; (2) explore the role of stepped geomorphology in shaping the present phylogeographic patterns; (3) infer the possible migration routes and dispersal corridors across the landscape of East Asia.

## Materials and Methods

### Sampling, DNA Extraction, PCR Amplification, and Sequencing

**Figure 1:**
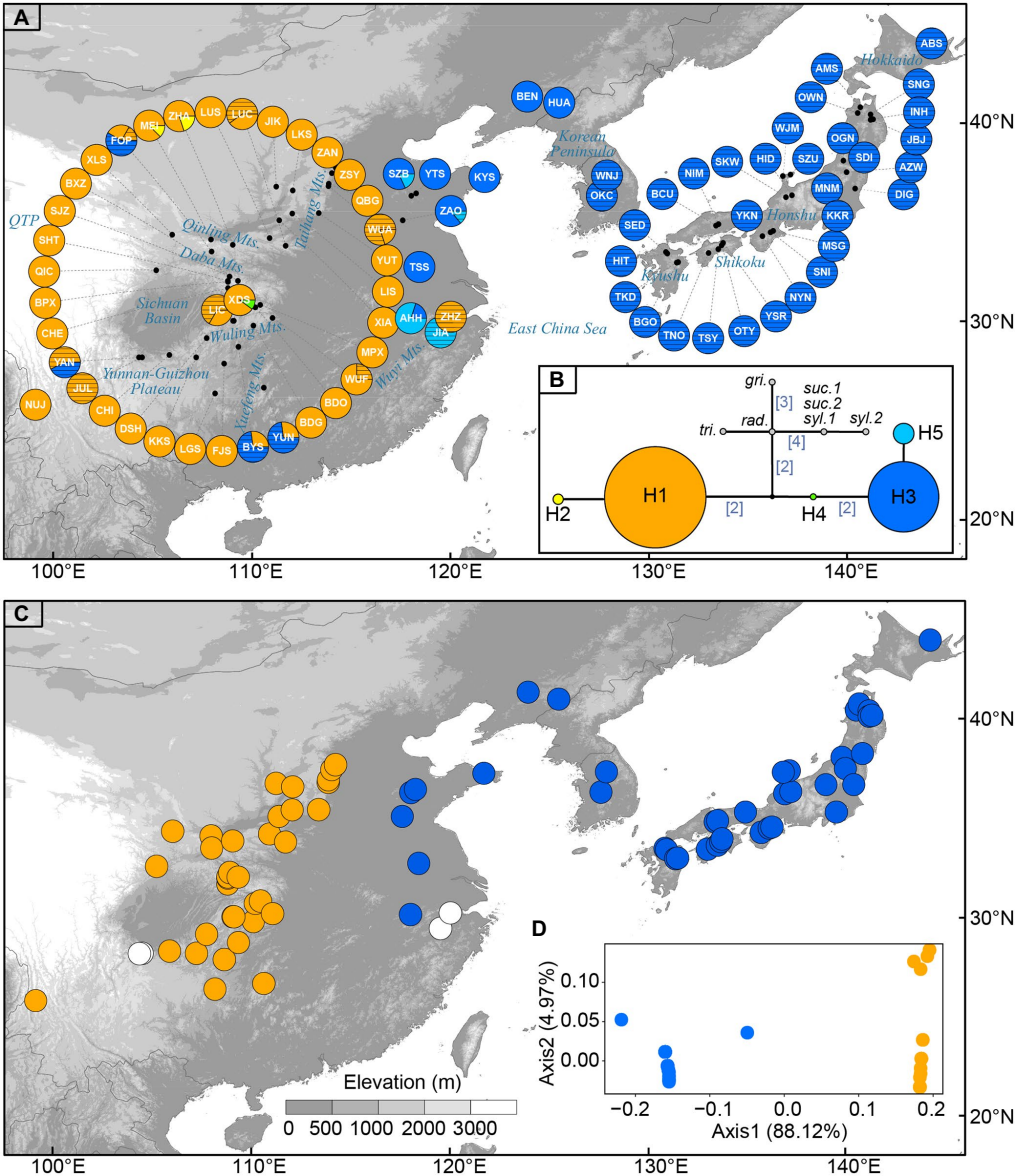
**(A)** Sampling sites of *Toxicodendron vernicifluum* in East Asia and geographic distribution of the five chloroplast (cp) haplotypes identified in this study. Each pie chart represents a sampling site (see Table 1 for site codes) and each haplotype is represented with a different color as shown in **(B)**. Sectors marked by black lines represent cultivated trees. (**B**) Median-joining network of cpDNA haplotypes estimated by POPART. Numbers in brackets on branches indicate the number of mutations between haplotypes when branches represent more than one mutation. Outgroups include *Toxicodendron griffithii* (*gri*.), *Toxicodendron radicans* (*rad.*), *Toxicodendron trichocarpum* (*tri.*), *Toxicodendron succedaneum* (*suc.*), and *Toxicodendron sylvestre* (*syl.*). **(C)** The western (orange) and eastern (blue) groups were identified by spatial analysis of molecular variance (SAMOVA) when *K* = 2. White circles represent sampling sites only including cultivated trees in mainland China. **(D)** Results of principal coordinate analysis (PCoA) based on the matrix of population pairwise *F*_ST_. Orange and blue circles represent the western and eastern groups, respectively.

Based on specimens and records in local flora, we selected 39 sampling sites that encompass the entire natural range of *T. vernicifluum* in China (Figure 1; Table 1). Between August 2018 and August 2021, we sampled leaf tissue from 357 individuals at these sites. Among those, 308 individuals from 36 sites were wild or semi-wild, while the remaining 49 individuals from seven sites were under cultivation (Table 1). At each site, three to 11 trees spaced >30 m apart were randomly sampled. Spatially explicit information was recorded for each tree using the 2bulu Outdoor Assistant app.^1^ Voucher specimens were deposited in the Herbarium of Nanjing Forestry University (NF).

**Table 1:** Locations and genetic statistics of 79 sampling sites of *Toxicodendron vernicifluum.*

Sampling sites	Location	Lon (E)	Lat (N)	E (m)	Wild trees				Cultivated trees	
					*n*_W_	Haplotypes	*H*_d_	*π*	*n*_C_	Haplotypes
*Western group*										
NUJ	Nujiang, Yunnan, China	99.10	25.78	2,204	10	H1(10)	0	0	0	–
YAN	Yanjin, Yunnan, China	104.32	28.16	997	0	–	–	–	7	H1(4), H3(3)
JUL	Junlian, Sichuan, China	104.48	28.19	393	0	–	–	–	10	H1(10)
QIC	Qingchuan, Sichuan, China	105.19	32.58	1,099	10	H1(10)	0	0	0	–
CHI	Chishui, Guizhou, China	105.86	28.29	1,271	3	H1(3)	0	0	0	–
KKS	Suiyang, Guizhou, China	107.20	28.18	1,242	10	H1(10)	0	0	0	–
DSH	Daozhen, Guizhou, China	107.73	29.14	1,304	10	H1(10)	0	0	0	–
LGS	Leishan, Guizhou, China	108.16	26.37	1,204	10	H1(10)	0	0	0	–
FJS	Jiangkou, Guizhou, China	108.61	27.87	1,005	10	H1(10)	0	0	0	–
BYS	Baojing, Hunan, China	109.33	28.71	436	3	H1(3)	0	0	8	H3(8)
BDG	Sangzhi, Hunan, China	110.09	29.78	1,336	10	H1(10)	0	0	0	–
YUN	Wugang, Hunan, China	110.61	26.65	1,166	3	H1(3)	0	0	8	H3(8)
LIC^*^	Lichuan, Hubei, China	109.06	30.03	829	1	H1(1)	–	–	2	H1(2)
XDS	Lichuan, Hubei, China	109.10	30.03	1,019	10	H1(9), H4(1)	0.200	0.00084	0	–
BDO^*^	Badong, Hubei, China	110.18	30.70	1,378	2	H1(2)	0	0	0	–
MPX	Zigui, Hubei, China	110.43	30.82	1,100	10	H1(10)	0	0	0	–
WUF^*^	Wufeng, Hubei, China	111.05	30.17	296	3	H1(3)	0	0	1	H1(1)
CHE^*^	Chengkou, Chongqing, China	108.80	31.67	2,294	6	H1(6)	0	0	0	–
BPX	Chengkou, Chongqing, China	108.80	31.99	1,140	10	H1(10)	0	0	0	–
XLS	Maiji, Gansu, China	106.00	34.36	1,397	10	H1(10)	0	0	0	–
MEI	Meixian, Shaanxi, China	107.95	34.12	692	8	H1(7), H2(1)	0.250	0.00035	0	–
FOP^**^	Foping, Shaanxi, China	107.98	33.51	895	3	H1(3)	0	0	9	H1(2), H3(7)
SHT	Langao, Shaanxi, China	108.80	32.05	2,409	10	H1(10)	0	0	0	–
SJZ	Langao, Shaanxi, China	108.88	32.27	741	10	H1(10)	0	0	0	–
ZHA	Zhashui, Shaanxi, China	109.05	33.86	1,178	10	H1(8), H2(2)	0.356	0.00050	0	–
BXZ	Pingli, Shaanxi, China	109.31	32.03	1,637	10	H1(10)	0	0	0	–
LUS	Lushi, Henan, China	110.90	34.21	1,194	10	H1(10)	0	0	0	–
LUC	Luanchuan, Henan, China	111.71	33.80	1,542	4	H1(4)	0	0	6	H1(6)
YUT	Xiuwu, Henan, China	113.37	35.45	1,016	5	H1(5)	0	0	0	–
JIK	Jiaokou, Shanxi, China	111.23	36.78	1,348	10	H1(10)	0	0	0	–
XIA	Xiaxian, Shanxi, China	111.39	35.09	936	10	H1(10)	0	0	0	–
LIS	Qinshui, Shanxi, China	112.05	35.43	1,510	10	H1(10)	0	0	0	–
LKS	Qinyuan, Shanxi, China	112.07	36.59	1,582	10	H1(10)	0	0	0	–
WUA^*^	Wu’an, Hebei, China	113.87	36.81	694	1	H1(1)	–	–	4	H1(4)
QBG	Wu’an, Hebei, China	113.88	36.94	752	10	H1(10)	0	0	0	–
ZSY	Zanhuang, Hebei, China	114.04	37.46	977	10	H1(10)	0	0	0	–
ZAN^*^	Zanhuang, Hebei, China	114.23	37.72	1,045	2	H1(2)	0	0	0	–
*Eastern group*										
AHH	Huangshan, Anhui, China	118.02	30.14	293	5	H3(1), H5(4)	0.400	0.00056	0	–
ZAO^**^	Zaozhuang, Shandong, China	117.52	35.11	229	12	H3(12)	0.264	0.00037	2	H5(2)
SZB	Boshan, Shandong, China	118.04	36.31	484	11	H3(9), H5(2)	0.327	0.00046	0	–
YTS^**^	Qingzhou, Shandong, China	118.28	36.46	599	11	H3(11)	0	0	0	–
KYS	Yantai, Shandong, China	121.73	37.27	209	7	H3(7)	0	0	0	–
BEN^**^	Benxi, Liaoning, China	123.87	41.32	269	14	H3(14)	0	0	0	–
HUA^*^	Huanren, Liaoning, China	125.49	41.02	230	3	H3(3)	0	0	0	–
TSS	Xuyi, Jiangsu, China	118.45	32.73	97	6	H3(6)	0	0	0	–
JIA^*^	Jiande, Zhejiang, China	119.52	29.44	47	0	–	–	–	4	H5(4)
ZHZ	Hangzhou, Zhejiang, China	120.03	30.22	48	0	–	–	–	3	H1(3)
OKC^*^	Okcheon, North Chungcheong, South Korea	127.64	36.33	194	0	–	–	–	4	H3(4)
WNJ^*^	Wonju, Gangwon, South Korea	127.91	37.35	156	0	–	–	–	2	H3(2)
SED^*^	Soeda, Fukuoka, Japan	130.87	33.49	249	0	–	–	–	1	H3(1)
HIT^*^	Hita, Oita, Japan	130.94	33.41	261	0	–	–	–	2	H3(2)
TKD^*^	Taketa, Oita, Japan	131.41	32.97	321	0	–	–	–	3	H3(3)
BGO^*^	Bungo-ono, Oita, Japan	131.49	32.99	175	0	–	–	–	1	H3(1)
TNO^*^	Tsuno, Kochi, Japan	133.02	33.45	494	0	–	–	–	2	H3(2)
TSY^*^	Kochi, Kochi, Japan	133.51	33.64	197	0	–	–	–	1	H3(1)
OTY^*^	Otoyo, Kochi, Japan	133.68	33.84	427	0	–	–	–	1	H3(1)
BCU^*^	Takahashi, Okayama, Japan	133.39	34.83	317	0	–	–	–	1	H3(1)
NIM^*^	Niimi, Okayama, Japan	133.52	34.89	281	0	–	–	–	2	H3(2)
YSR^*^	Miyoshi, Tokushima, Japan	133.75	33.96	216	0	–	–	–	1	H3(1)
YKN^*^	Fukuchiyama, Kyoto, Japan	134.94	35.32	154	0	–	–	–	1	H3(1)
NYN^*^	Gojo, Nara, Japan	135.73	34.29	193	0	–	–	–	2	H3(2)
SNI^*^	Soni, Nara, Japan	136.14	34.50	592	0	–	–	–	2	H3(2)
MSG^*^	Tsu, Mie, Japan	136.27	34.55	227	0	–	–	–	2	H3(2)
SKW^*^	Shirakawa, Gifu, Japan	136.91	36.26	570	0	–	–	–	1	H3(1)
HID^*^	Hida, Gifu, Japan	137.22	36.34	949	0	–	–	–	2	H3(2)
WJM^*^	Wajima, Ishikawa, Japan	136.89	37.33	123	0	–	–	–	3	H3(3)
SZU^*^	Suzu, Ishikawa, Japan	137.14	37.40	213	0	–	–	–	1	H3(1)
MNM^*^	Minakami, Gunma, Japan	138.99	36.70	516	0	–	–	–	1	H3(1)
KKR^*^	Kamakura, Kanagawa, Japan	139.51	35.31	14	0	–	–	–	1	H3(1)
OGN^*^	Oguni, Yamagata, Japan	139.81	38.09	209	0	–	–	–	2	H3(2)
AZW^*^	Aizuwakamatsu, Fukushima, Japan	139.97	37.51	405	0	–	–	–	1	H3(1)
DIG^*^	Daigo, Ibaraki, Japan	140.40	36.70	145	0	–	–	–	1	H3(1)
OWN^*^	Owani, Aomori, Japan	140.53	40.49	121	0	–	–	–	1	H3(1)
AMS^*^	Aomori, Aomori, Japan	140.67	40.78	76	0	–	–	–	2	H3(2)
SNG^*^	Shingo, Aomori, Japan	141.18	40.43	158	0	–	–	–	1	H3(1)
SDI^*^	Sendai, Miyagi, Japan	140.85	38.26	59	0	–	–	–	1	H3(1)
JBJ^*^	Ninohe, Iwate, Japan	141.18	40.16	435	0	–	–	–	1	H3(1)
INH^*^	Ichinohe, Iwate, Japan	141.31	40.20	197	0	–	–	–	3	H3(3)
ABS^*^	Abashiri, Hokkaido, Japan	144.25	44.01	145	0	–	–	–	2	H3(2)

Lon, longitude; Lat, latitude; E, elevation; *n*_W_, number of wild trees; *H*_d_, haplotype diversity; π, nucleotide diversity; *n*_C_, number of cultivated trees.

* Sequences were obtained by Suzuki et al. (2014);

** Sequences were obtained by both Suzuki et al. (2014) and this study.

Total genomic DNA was extracted from silica-gel dried leaf material according to the manufacturer’s protocol for the Plant Genomic DNA Kit (Tiangen, Beijing, China). The concentration of DNA samples was diluted to 10 ng/μl and stored at −20°C for PCR amplification. Following Suzuki et al. (2014), we used two chloroplast intergenic spacers (*trnL* and *trnL-F*) to sequence all the 357 samples. Polymerase chain reactions (PCRs) were performed using a Mastercycler pro Thermal Cycler (Eppendorf, Germany) in 25-μl reaction volumes as described by Zhang et al. (2015). Thermal cycling started with a denaturation step lasting 10 min at 95°C, followed by 30 cycles each comprising 30 s of denaturation at 94°C, 40 s of annealing at 50°C, and 60 s of elongation at 72°C. Amplification ended with a 10-min extension at 72°C. The PCR products were purified and sequenced using the ABI 3730XL DNA Analyzer by Sangon Biotech (Shanghai, China).

We compiled previously published sequence data of the same two cpDNA regions for 92 *T. vernicifluum* trees sampled from 30 sites in Japan (*n* = 46 trees), two sites in South Korea (*n* = 6 trees), and 12 sites in China (*n* = 40 trees; Suzuki et al., 2014). Among those, all the Japanese and Korean samples were collected from cultivated trees, while 15 (from six sites) and 25 (from 10 sites) Chinese samples were collected from cultivated and wild trees, respectively. Given that four sites in Suzuki et al. (2014) were close to our sampling locations (i.e., FOP, ZAO, YTS, and BEN), the corresponding sequence data were combined for each site. Finally, we obtained a dataset for 449 *T. vernicifluum* trees (333 wild trees and 116 cultivated trees) sampled from 79 sites, including 397 trees from 47 sites in China, 46 trees from 30 sites in Japan, and six trees from two sites in South Korea (Table 1). Furthermore, we also compiled the sequence data for five congeners that were used as outgroups, including *T. succedaneum* (Suzuki et al., 2014; Wang et al., 2020b), *T. sylvestre* (He et al., 2020), *T. trichocarpum* (Suzuki et al., 2014), *T. radicans* (Suzuki et al., 2014), and *T. griffithii* (Li et al., 2020). GenBank accession numbers for all the samples analyzed in this study were listed in Supplementary Table S1.

### Chloroplast Sequence Data Analyses

All sequences were checked and aligned by BioEdit 7.2.5 (Hall, 1999). The obtained alignments were concatenated into a single matrix using FasParser 2.1.1 (Sun, 2017). A 96-bp indel detected in the *trnL-F* region was treated as a single mutation event and coded as a substitution (A/T). Chloroplast haplotypes were determined by DnaSP 5.10 (Librado and Rozas, 2009). A median-joining network was inferred with PopART 1.7 to visualize the phylogenetic relationships among haplotypes (Leigh and Bryant, 2015). ArcGIS 10.5 was employed to show the geographic distribution of haplotypes across the range of *T. vernicifluum* in East Asia.

We calculated haplotype diversity (*H*_d_) and nucleotide diversity (*π*) for each sampling site using DnaSP. We estimated average gene diversity within sampling sites (*h*_S_), total gene diversity (*h*_T_), and two genetic differentiation coefficients *G*_ST_ and *N*_ST_ using Permut 2.0 (Pons and Petit, 1996). *N*_ST_ is a measure of genetic differentiation among sites considering genetic distances between haplotypes, whereas *G*_ST_ is an unordered measure that does not take distances among haplotypes into account. A higher *N*_ST_ than *G*_ST_ usually indicates the presence of a phylogeographic structure, i.e., closely related haplotypes are more frequently observed in the same populations than less related ones (Pons and Petit, 1996). The significance of the difference between *G*_ST_ and *N*_ST_ was tested by a permutation test (*n* = 10,000). In these analyses, we excluded the cultivated trees in China but included those in Japan and South Korea because they represent the distribution of *T. vernicifluum* outside China and may have a natural origin in mainland China. Given that Permut requires a minimum sample size of three for each sampling site, we first combined the adjacent sites (*n* < 3) within the same 0.625° × 0.625° grid into one site and then removed the sites still with less than three wild trees. Finally, 10 sites in Japan were combined into five sites and 27 sites (including eight sites in China, one site in South Korea, and 18 sites in Japan) were excluded, resulting in a dataset comprising 353 individuals from 47 sites (including 327 trees from 39 sites in China, four trees from one site in South Korea, and 22 trees from seven sites in Japan; Supplementary Table S2).

To examine the phylogeographic structure of *T. vernicifluum*, we performed a spatial analysis of molecular variance (SAMOVA) using the software SAMOVA 2.0 (Dupanloup et al., 2002). This analysis used a simulated annealing procedure to maximize the proportion of total genetic variance (*F*_CT_) due to differences between groups of populations. One hundred independent runs were carried out for each number of groups (*K*) ranging from 2 to 10 to ensure that the final configuration of the *K* groups is not affected by a given initial configuration. For the most likely *K*, the significance of variance components (overall genetic variance partitioned among groups, among populations within groups, and within populations) and their associated fixation indices (*F*_CT_, *F*_SC_, and *F*_ST_) was assessed by 10,000 random permutations using Arlequin 3.5 (Excoffier et al., 2005). To validate the results of SAMOVA, we also performed principal coordinate analysis (PCoA) based on the matrix of population pairwise *F*_ST_ using GenALEx 6.5 (Peakall and Smouse, 2012). To test for the isolation by distance (IBD) pattern, we examined the correlation between population pairwise *F*_ST_ and the logarithm of geographic distance (km) using a Mantel test. The significance of the correlation was assessed by 9,999 permutations in GenALEx 6.5. In these analyses, we only removed the cultivated trees in China for the reason mentioned above. The final dataset comprised 385 trees from 75 sampling sites, including 333 trees from 43 sites in China, six trees from two sites in South Korea, and 46 trees from 30 sites in Japan (Supplementary Table S2). We used BEAST 2.6.7 (Bouckaert et al., 2019) to estimate the divergence time between the eastern and western clades of *T. vernicifluum*. The phylogenetically closest species in the median-joining network, *T. radicans*, was used as an outgroup. The best-fitting substitution model HKY was selected by ModelFinder (Kalyaanamoorthy et al., 2017). A combination of strict clock and Bayes-skyline coalescent prior was used for node age estimation. The mean value of cpDNA substitution rate for angiosperms (2.0 × 10^−9^ substitutions per site per year) was employed (Wolfe et al., 1987; Sakaguchi et al., 2012). Two independent MCMC runs were performed for 100 million generations and sampled every 10,000 generations. Tree and log files were combined through LogCombiner 2.6.7, and then passed to Tracer 1.7.1 (Rambaut et al., 2018) for assessing convergence, and to TreeAnnotator 2.6.7 for constructing a maximum clade credibility tree with a posterior probability limit of 0.5 and the first 20% generations discarded as burn-in.

### Ecological Niche Modeling and Niche Identity Test

We used the maximum-entropy approach in Maxent 3.4.1 (Phillips et al., 2018) to model the present distribution of *T. vernicifluum* and to reconstruct its potential distribution during the Last Interglacial [LIG; ~0.12–0.14 million years ago (mya)] and the Last Glacial Maximum (LGM; ~0.022 mya). Species occurrence data were obtained from five sources: the Global Biodiversity Information Facility (GBIF),^2^ the Chinese Virtual Herbarium (CVH),^3^ the Plant Photo Bank of China (PPBC),^4^ literature (Wei et al., 2010; Bai et al., 2017; Guo et al., 2019), and field investigation. The Getpoint tool of Baidu Maps^5^ was used to collect coordinates for the specimen records only with explicit locality information. We filtered our dataset by removing duplicate records and retaining only one observation within each 2.5′ × 2.5′ grid to reduce the effect of spatial autocorrelation. Finally, a total of 394 presence points were obtained, of which 307, 32, and 46 records were from China, Japan, and South Korea, respectively. We retrieved the climatic data for the present and LGM from the WorldClim 1.4 database^6^ at a spatial resolution of 2.5′. The LGM data were generated based on the outputs of the Community Climate System Model 4 (CCSM4). The raster layers of the LIG were obtained from the WorldClim 1.4 at a spatial resolution of 30″ and then resampled to 2.5′ *via* the nearest neighbor method as implemented in ArcGIS 10.5. We eliminated highly correlated variables (|Pearson’s *r*| ≥ 0.8) to prevent potential over-fitting. Finally, six of the 19 bioclimatic variables (Supplementary Table S3) provided by the WorldClim database were retained, including annual mean temperature (bio1), mean diurnal range (bio2; the mean of the difference of the monthly maximum and minimum temperatures over a year), isothermality (bio3), temperature seasonality (bio4), mean temperature of the wettest quarter (bio8), and annual precipitation (bio12). All the environmental layers were clipped to the same spatial range (15°–45°N, 90°–145°E) using the package “raster” 2.8-19 (Hijmans, 2019) in R 3.6.0 (R Core Team, 2018). We ran the Maxent model with default settings. Model performance was assessed using the areas under the receiver operating characteristic curve (AUC) produced by 10-fold cross-validation. The generated model was projected onto the two historical periods and the predicted suitable areas were visualized based on the logistic outputs of Maxent using ArcGIS 10.5. Principal component analysis (PCA) was performed with the 19 bioclimatic variables provided by the WorldClim database for all the presence points of *T. vernicifluum* using R 3.6.0.

We used ENMTools (Warren et al., 2010) to quantify the niche overlap between the SDMs generated for the western and eastern populations of *T. vernicifluum*. Two statistics, Schoener’s *D* (Schoener, 1968) and Warren’s *I* (Warren et al., 2008), were used to measure the niche overlap. These two indices are limited between 0 (the two groups have a completely discordant niche) and 1 (the two groups have an identical niche). To perform the niche identity test, first, we used the same program to create a pseudoreplicate dataset by randomly partitioning the pooled occurrence points for the two groups into two new sub-datasets with the original sample size (i.e., 457 and 54). Then, the new dataset was imported to Maxent to generate new SDMs using the default settings. Finally, we calculate Schoener’s *D* and Warren’s *I* for SDMs generated by 100 pseudoreplicate datasets. The niche identity was tested by comparing the observed values and the null distributions for these two statistics.

### Migration Vector Analysis and Dispersal Corridors

To visualize the migration direction of *T. vernicifluum* between different periods, we performed a migration vector analysis following Gugger et al. (2013). First, the logistic outputs of Maxent were converted into presence/absence maps using the ‘maximum test sensitivity plus specificity’ threshold (Jiménez-Valverde and Lobo, 2007). Second, we estimated the geographic centroids of all the 0.625° × 0.625° grids (i.e., each grid contained 15 × 15 grid cells in 2.5′) for each period using the zonal geometry function in ArcGIS 10.5. Finally, we inferred migration vectors by seeking the nearest centroids of the second period to each centroid of the first period using ArcGIS 10.5 (i.e., from LIG to LGM and from LGM to present). The obtained maps showed the potential population sources from one period to the next.

We also integrated SDMs and shared haplotype information to infer the putative dispersal corridors across the landscape (Chan et al., 2011). First, we inverted the logistic outputs of Maxent (*x* inverted = 1−*x*) to create a friction layer (i.e., a dispersal cost layer), which depicted the ease of dispersal from each locality through the landscape. Second, we calculated a single least-cost path (LCP) and multiple least-cost corridors (LCCs) for each pair of sampling sites that shared haplotypes (Graves et al., 2014; Yu et al., 2015). Least-cost corridors were classified into three categories according to the percentage by which the path length was greater than that of the LCP: low (<1.0%), mid (<2%), and high (<5%). Finally, we summed all the LCCs to create a raster of the dispersal network. The low, mid, and high classes were weighted by 5, 2, and 1, respectively. Dispersal corridors were expected to be the areas where LCCs traversed more frequently. In this analysis, we removed the records of cultivated trees in mainland China, including four sampling sites (i.e., YAN, JUL, JIA, and ZHZ) that were only composed of cultivated trees. Furthermore, only one site was chosen within each 0.625° × 0.625° grid to reduce both computational cost and sampling bias. Finally, a total of 62 localities (Supplementary Table S2) were retained to infer the dispersal corridors of *T. vernicifluum* during the present, LGM, and LIG using SDMtoolbox 2.0 (Brown, 2014).

## Results

### Phylogenetic Relationship and Geographic Distribution of Chloroplast Haplotypes

The combination of the *trnL* and *trnL-F* datasets resulted in an alignment with a total length of 717 bp. Based on seven substitutions, five chloroplast haplotypes (H1–H5) were identified across the 79 sampling sites of *T. vernicifluum* (Supplementary Table S4). The median-joining network grouped the haplotypes into two major lineages separated by three mutational steps (Figure 1B). Among those, the western clade was mainly detected in western China, including two haplotypes H1 and H2, while the eastern clade was mostly distributed in eastern China, South Korea, and Japan, comprising three haplotypes H3, H4, and H5 (Figure 1A). Exceptions were the natural occurrence of H4 in one western China site XDS, and the introduction of the trees with H3 to western China (i.e., sites JUL, BYS, YUN, and FOP) and those with H1 to eastern China (i.e., site ZHZ; Figure 1A).

Among the five haplotypes, H1 and H3 were at the interior of the network and were represented by 65.0 and 31.4% of the surveyed samples, respectively (Figure 1B). Wild trees with H1 were detected in all the 37 sampling sites of western China, while the haplotype H3 was observed in 40 of the 42 sites in eastern China, South Korea, and Japan. In contrast, H2 and H5 were found at the tips of the network, separated from H1 or H3 by one mutational step (Figure 1B). They had a much lower frequency, of which H2 was only detected in two sampling sites (i.e., MEI and ZHA) located at the Qinling Mountains, western China, and H3 occurred in four sites of eastern China (i.e., ZAO, SZB, AHH, and JIA). The haplotype H4 was found at an intermediate position between H1 and H3, represented by only one individual in the sampling site XDS. All five haplotypes were detected in mainland China. Most Chinese sites only had one haplotype, while 10 sites (i.e., YAN, FOP, MEI, ZHA, XDS, BYS, YUN, AHH, ZAO, and SZB) had two haplotypes. All the Korean and Japanese sites were fixed for the haplotype H3 (Figure 1A).

### Chloroplast DNA Diversity, Differentiation, and Phylogeographic Structure

When the cultivated trees in mainland China were excluded, the number of haplotype, haplotype diversity (*H*_d_), and nucleotide diversity (*π*) of each sampling site ranged from 1 to 2 (mean = 1.127), 0 to 0.4 (mean = 0.042), and 0 to 0.00084 (mean = 0.00007), respectively (Table 1). The highest level of haplotype diversity was observed in AHH (*H*_d_ = 0.4), followed by ZHA (*H*_d_ = 0.356) and SZB (*H*_d_ = 0.327), while the highest level of nucleotide diversity was observed in XDS (*π* = 0.00084), followed by AHH (*π* = 0.00056), and ZHA (*π* = 0.0005). The total gene diversity (*h*_T_ = 0.484 ± 0.049) across all sampling sites was found to be much higher than average gene diversity within sites (*h*_S_ = 0.033 ± 0.014; Table 2).

**Table 2:** Genetic statistics of *Toxicodendron vernicifluum* based on the sequence variation at two chloroplast (cp) DNA markers.

Sampling sites	Sample size	*h*_T_ (SE)	*h*_S_ (SE)	*G*_ST_ (SE)	*N*_ST_ (SE)	*p*-Value
Sites in mainland China	327	0.361 (0.081)	0.039 (0.017)	0.891 (0.044)	0.972 (0.013)	0.027^*^
Sites across the species’ range	353	0.484 (0.049)	0.033 (0.014)	0.933 (0.028)	0.983 (0.008)	0.029^*^

The samples used in these analyses are described in Supplementary Table S2. *h*_S_, average gene diversity within sampling sites; *h*_T_, total gene diversity; *G*_ST_, genetic differentiation at the two cpDNA markers; *N*_ST_, genetic differentiation at the two cpDNA markers taking similarities between haplotypes into account; SE, standard error.

* *p* < 0.05, indicating that *N*_ST_ is significantly larger than *G*_ST_.

Genetic differentiation among sampling sites was substantial as indicated by the high values of *G*_ST_ and *N*_ST_. Comparisons of these two measures showed that a significant phylogeographic structure occurred across the species’ range (*N*_ST_ = 0.983 > *G*_ST_ = 0.933; *p* = 0.029) or across the sampling sites in mainland China (*N*_ST_ = 0.972 > *G*_ST_ = 0.891; *p* = 0.027; Table 2). SAMOVA revealed a high level of differentiation among groups (*F*_CT_ > 0.980) for all the *K* values from 2 to 10. When *K* = 2, the sampling sites of *T. vernicifluum* were divided into two groups (Figure 1C). The western group included the 35 sites in western China, while the eastern group included the 40 sites in eastern China, South Korea, and Japan. These two groups were separated clearly by the boundary between the middle and low units of the three-step landforms of China. Hierarchical AMOVA showed that 98.78% of the total genetic variance was partitioned between these two groups (*F*_CT_ = 0.988, *p* = 0.000), while only 0.24% and 0.97% of the variance were partitioned among sampling sites within groups (*F*_SC_ = 0.199, *p* = 0.042) and within sites (*F*_ST_ = 0.990, *p* = 0.000), respectively (Table 3). PCoA obtained a result consistent with that of SAMOVA (*K* = 2). The first axis explained 88.12% of the total variance (Figure 1D). No overlap along this axis was detected between the individuals of the western and eastern groups. IBD analyses showed that the geographic isolation effect was significant across all sampling sites of *T. vernicifluum* (*R* = 0.682, *p* = 0.001) or across those in mainland China (*R* = 0.610, *p* = 0.000). The BEAST analysis also grouped the haplotypes into western and eastern clades (posterior probability = 1). The divergence time between these two clades was estimated to be 1.36 mya (95% HPD; 0.51–2.41 mya; Supplementary Figure S1).

**Table 3:** Hierarchical analyses of molecular variance (AMOVAs) based on chloroplast (cp) DNA haplotype frequencies of *Toxicodendron vernicifluum.*

Source of variation	df	SS	VC	Variation (%)	Fixation index
Among groups	1	413.19	2.49	98.78	*F*_CT_ = 0.988^**^
Among populations within groups	73	4.05	0.01	0.24	*F*_SC_ = 0.199^*^
Within populations	310	7.61	0.02	0.97	*F*_ST_ = 0.990^**^

The samples used in this analysis are described in Supplementary Table S2. df, degree of freedom; SS, sum of squares; VC, variance components. *p*-value was obtained through 10,000 permutations in ARLEQUIN.

** *p* < 0.01;

* *p* < 0.05.

### Ecological Niche Modeling and Niche Identity Test

**Figure 2:**
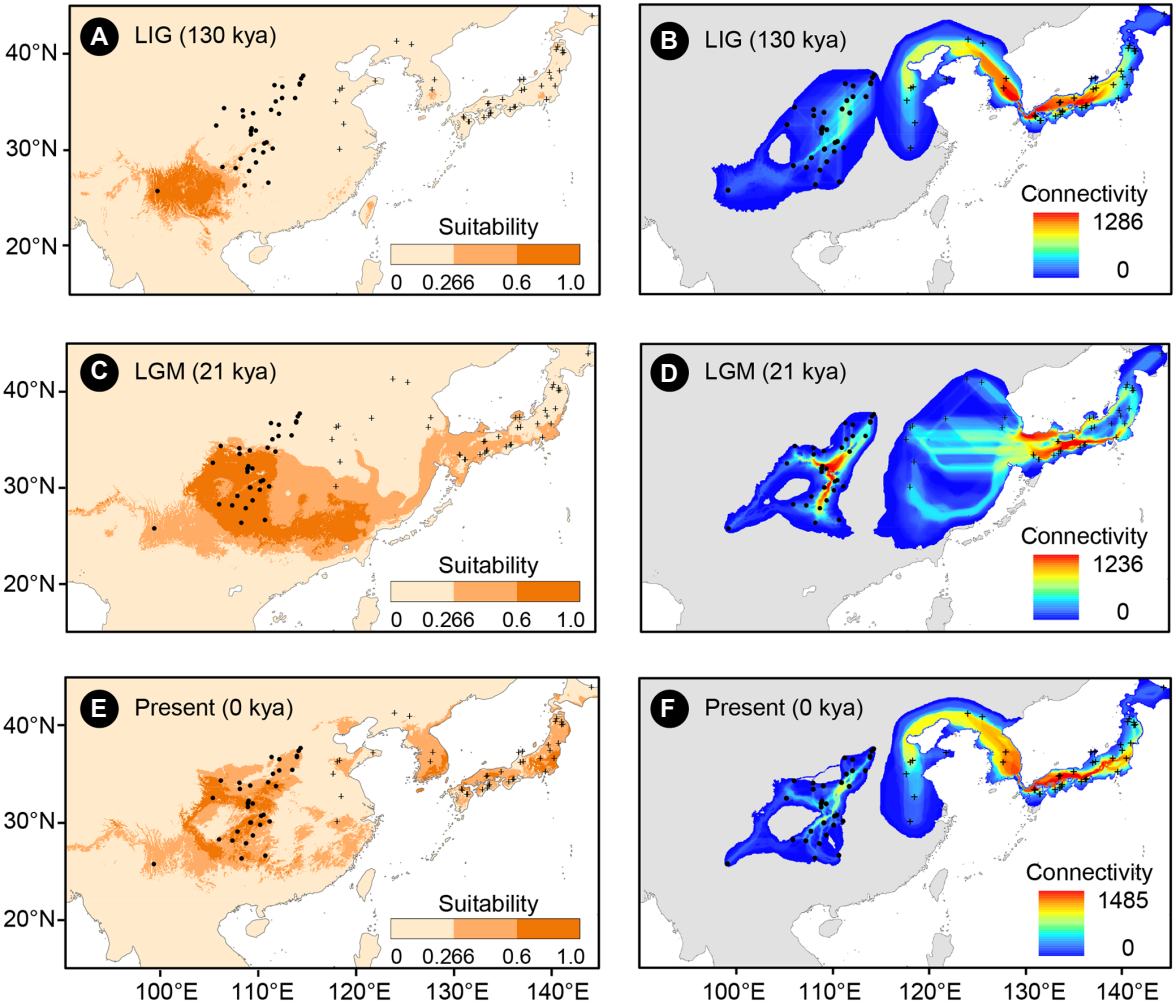
Climatically suitable areas and dispersal corridors of *Toxicodendron vernicifluum* during the Last Interglacial (LIG; **A,B**), Last Glacial Maximum (LGM; **C,D**), and the present (**E,F**) based on the outputs of ecological niche modeling (ENM) using Maxent 3.4.1 (Phillips et al., 2018). Black dots and plus signs represent the sampling sites of the western and eastern groups, respectively.

**Figure 3:**
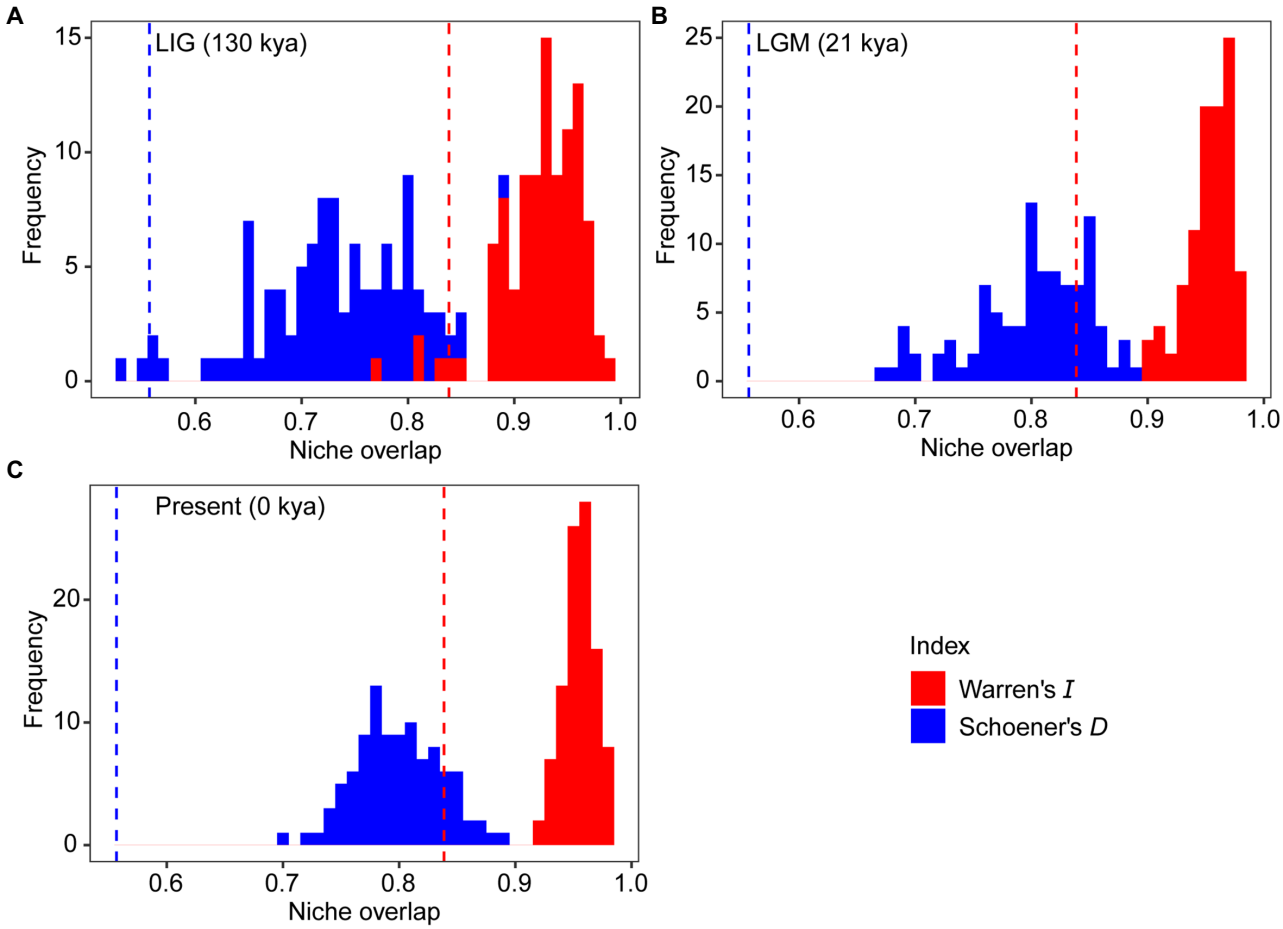
Niche divergence between the western and eastern groups of *Toxicodendron vernicifluum* during the Last Interglacial (LIG; **A**), Last Glacial Maximum (LGM; **B**), and present **(C)**. The niche identity test was conducted to quantify niche overlap using two indices, Warren’s *I* (red) and Schoener’s *D* (blue). Vertical lines and histograms represent the observed values and null distributions (based on 100 pseudoreplicates) of niche overlap statistics, respectively.

**Figure 4:**
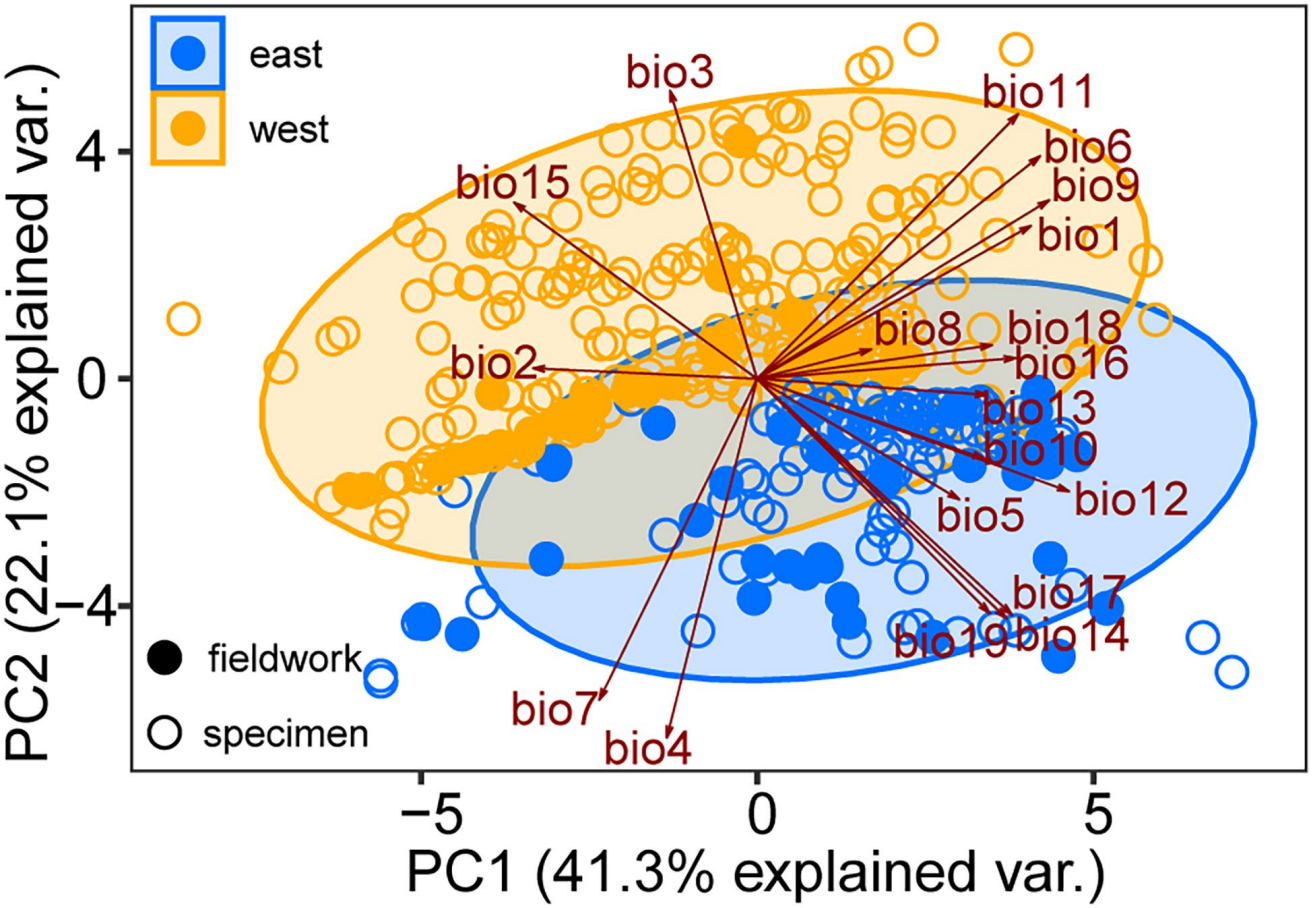
Principal component analysis (PCA) performed with 19 bioclimatic variables (Supplementary Table S3) for the specimen records (open dots) and sampling sites (solid dots) of *Toxicodendron vernicifluum*. Presence points of the western and eastern groups are marked in orange and blue colors, respectively.

The mean AUC value (±SD) was 0.895 ± 0.009, indicating that the SDM fitted well with the observed dataset. The predicted potential distributions of *T. vernicifluum* were shown in Figure 2. At present, the suitable habitats of *T. vernicifluum* are mainly distributed in western China, the Korean Peninsula, and Japan, encompassing a large number of the presence points (Figure 2E). The highly suitable habitats in China are mainly found in the mountainous areas of the “middle step” region, including the Yunnan-Guizhou Plateau, Qinling Mountains, and Daba mountains. During the LGM, the suitable habitats were widely distributed in western and southern China and extended to the offshore areas of the East China Sea Shelf and Japan (Figure 2C). During the LIG, the suitable areas were mainly restricted to southwestern China (Figure 2A). Both Schoener’s *D* and Warren’s *I* indicated that significant climatic niche divergence occurred between the western and eastern groups of *T. vernicifluum*, regardless of whether the sampling sites of South Korea and Japan were included (Schoener’s *D* = 0.557, *p* = 0.000; Warren’s *I* = 0.839, *p* = 0.000) or not (Schoener’s *D* = 0.472, *p* = 0.000; Warren’s *I* = 0.756, *p* = 0.000; Figure 3). Similar results were also obtained for the LIG and LGM (Figure 3). PCA plot showed that the western group was associated with higher isothermality, higher precipitation seasonality, lower precipitation of the driest month, lower precipitation of the driest quarter, and lower precipitation of the coldest quarter (Figure 4).

### Migration Vector Analysis and Dispersal Corridors

**Figure 5:**
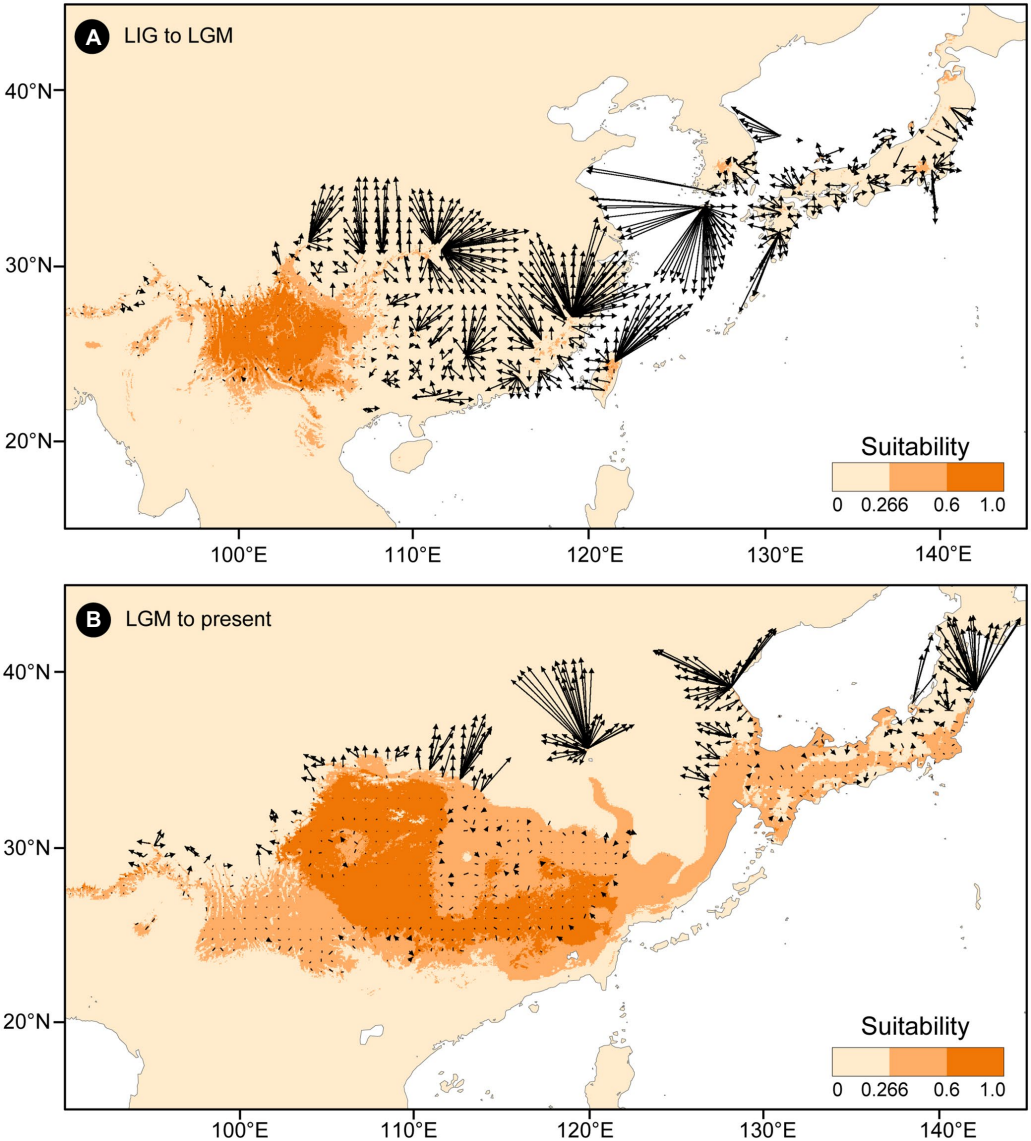
Migration vector analysis of local changes in climatically suitable areas of *Toxicodendron vernicifluum* between the Last Interglacial (LIG) and Last Glacial Maximum (LGM; **A**), and between the LGM and present **(B)**. Black arrows represent the potential migration direction from one period to the next.

The putative migration directions of *T. vernicifluum* between adjacent periods were shown in Figure 5. Suitable areas were inferred to have expanded continuously since the LIG. From the LIG to LGM, the suitable areas in southwestern China may have expanded northward or eastward to the mountainous areas in central China (e.g., the Qinling Mountains and the Funiu Mountains). Notably, some scattered suitable habitats in southern and southeastern China (e.g., the Nanling Mountains and the Wuyi Mountains) may have also expanded northward drastically. Furthermore, both the suitable areas in the Taiwan Island and the Korean Peninsula may have extended to the East China Sea Shelf during the LGM. From the LGM to the present, *T. vernicifluum* was predicted to have spread further northward. They may have migrated from the Funiu Mountains to the Taihang Mountains, from the Shandong and Korean Peninsulas to northeastern China, and from central Japan to Hokkaido.

No population connectivity was found between western and eastern China because the wild trees of western and eastern groups did not share any haplotypes (Figure 2). Instead, two major dispersal routes were identified in the eastern and western parts of the range of *T. vernicifluum*. High landscape connectivity was detected among populations in northeastern China, the Korean Peninsula, and Japan. The ECS land bridge may have contributed to the migration to Japan during the LGM. In western China, dispersal corridors occurred around the mountain ranges east of the Sichuan Basin, including the Taihang Mountains, the Qinling Mountains, the Wu Mountain, and the Xuefeng Mountains (Figure 2).

## Discussion

### East-West Phylogeographic Split Associated With the Stepped Geomorphology of China

Our chloroplast DNA analyses support an east-west phylogeographic split of *T. vernicifluum* (Figure 1). The two recognized clades were separated according to the stepped landforms of mainland China, with the wild individuals of the western clade geographically restricted to the “middle step”, while those of the eastern clade mainly confined to the “low step”. The two “steps” differ in geomorphology; the “middle step” is characterized by a vast extent of mountains and plateaus (average ~2,000 m), while the “low step” is mainly made up of hills and plains (average <500 m; Jiang and Wu, 1993; Wan, 2012). Moreover, a series of northeast-southwest oriented mountain ranges (e.g., the Taihang Mountains, the Wu Mountains, and the Xuefeng Mountains) occur on the border between the two “steps”, further increasing the ruggedness of local terrain (Jiang and Wu, 1993; Li et al., 2015). The existence of these long-standing geographic barriers, together with the extreme physiographical heterogeneity in mainland China, may have strongly restricted the dispersal of *T. vernicifluum* across the landscape, leading to long-term isolation and allopatric genetic divergence between western and eastern lineages of *T. vernicifluum* (Qian and Ricklefs, 2000; Zhang et al., 2018; Luo et al., 2021).

We inferred that the separation between the two major lineages may have occurred during the Early Pleistocene (1.36 mya, 95% HPD: 0.51–2.41 mya; Supplementary Figure S1). This timing is comparable with the intraspecific divergence date of *Quercus acutissima* (1.31 mya; 95% HPD: 1.27–1.34 mya; Gao et al., 2021), a species also exhibiting an east-west break associated with the stepped landforms of China. These results suggest that they may have undergone a similar biogeographic history affected by the landscape features in East Asia and climatic oscillations during the Early Pleistocene. The cool climate may have caused the ancestral population to retreat to different refugia, leading to allopatric divergence in response to long-term geographic isolation. At present, the western clade of *T. vernicifluum* was mainly observed in highlands (between 800 and 2,000 m) with higher isothermality and lower precipitation of the driest quarter, while the eastern clade usually occurs in lowlands (less than 600 m) with opposite climatic conditions (Figure 4). The climate niche divergence was estimated to have occurred no later than the LIG (Figure 3), suggesting that local adaptation may have also contributed to the splitting of *T. vernicifluum*. However, our dating results must be interpreted with extreme caution because of the wide range of divergence time and uncertainty in mutation rate (Luo et al., 2021; Li et al., 2022). Nonetheless, our data depict the most likely scenario that the two major clades have diverged before the LGM, which would provide more opportunities for those lineages to adapt to distinct climatic conditions.

Previous studies have revealed an east-west phylogeographic break across the boundary between the middle and low “steps” in three widespread woody plants, including *K. septemlobus* (Sakaguchi et al., 2012), *K. japonica* (Luo et al., 2021), and *Q. acutissima* (Zhang et al., 2018). For the two former species, the pattern was confirmed by both nuclear microsatellite and chloroplast sequence markers, although limited admixture was detected near the border (Sakaguchi et al., 2012; Luo et al., 2021). For the last species, the pattern was revealed only by nuclear microsatellites (Zhang et al., 2018). A high level of genetic admixture was observed in central China, probably because wind-pollinated oak species have greater long-distance pollen-mediated gene flow than the other two insect-pollinated plants (Buschbom et al., 2011). In subtropical China, similar patterns with limited sharing of genetic variation between the two “step” were observed in several woody and herbaceous plants, such as *J. cathayensis* (Bai et al., 2014), *Castanopsis eyrei* (Shi et al., 2014), *Castanopsis fargesii* (Sun et al., 2014), *Cyclocarya paliurus* (Kou et al., 2016), *B. clarkeana* (Wang et al., 2018), and *L. chinense* (Yang et al., 2019; Zhong et al., 2019). In comparison with those species, *T. vernicifluum* exhibits a much sharper east-west phylogeographic break; no haplotypes were found to be shared among wild populations from the two sides (Figure 1A). Furthermore, the break not only occurs in subtropical areas (e.g., across the Xuefeng Mountain and the Wu Mountain) but also extends to warm-temperate regions that were not fully covered by previous studies, such as the Taihang Mountains (Figure 1A).

Another remarkable feature of the present phylogeographic pattern is that more than 90% of the sampling sites are fixed for a single haplotype (Figure 1A). The near-complete fixation of two major haplotypes (H1 and H3) in western vs. eastern China resulted in an extremely low level of genetic diversity within populations (*h*_S_ = 0.033) and an extremely high level of genetic differentiation among populations (*G*_ST_ = 0.933; Table 2). Such patterns are comparable with those of previously mentioned subtropical plants such as *J. cathayensis* (*h*_S_ = 0, *G*_ST_ = 1; Bai et al., 2014), *C. paliurus* (*h*_S_ = 0.066, *G*_ST_ = 0.924; Kou et al., 2016), and *B. clarkeana* (*h*_S_ = 0.030, *G*_ST_ = 0.964; Wang et al., 2018), suggesting that they have experienced a common phylogeographic history of long-term isolation across fragmented mountainous habitats in subtropical China (Wang et al., 2009; Du et al., 2011; Bai et al., 2014; Li et al., 2019). *Toxicodendron vernicifluum* prefers to grow in highlands and thus exhibits a scattered distribution in mountainous areas. Within a separated region, strong forces of genetic drift, combined with low mutation and low migration rates of cpDNA sequences, would greatly reduce the genetic diversity within populations (Bai et al., 2014). Furthermore, as a dioecious plant, *T. vernicifluum* is predicted to be more sensitive to genetic drift and tend to show a higher level of differentiation as it has a smaller effective population size than hermaphrodite species (McCauley, 1994). This prediction has been verified in a dioecious shrub, *Ilex aquifolium*, which exhibited twice the level of differentiation in comparison with hermaphrodite species investigated over a similar geographical scale (Rendell and Ennos, 2003). Overall, we suggest that both abiotic (e.g., long-term geographic isolation) and biotic (e.g., a dioecious breeding system) factors have contributed to the occurrence of a pronounced phylogeographic structure in *T. vernicifluum*.

### Glacial Refugia, Historical Migration, and Human-Aided Dispersal

The present phylogeographic structure of *T. vernicifluum* suggests that the species has experienced long-term isolation between at least two refugia in western and eastern China (Bai et al., 2014; Liao et al., 2014). However, the exact locations of refugia are uncertain because each of the two areas is dominated by an ancestral haplotype (H1 and H3) that exhibits a much wider distribution than others (Posada and Crandall, 2001). Combining the prediction of ENMs, we infer that the mountainous areas in southwestern (i.e., the Yunnan-Guizhou Plateau) and southeastern China are more likely to be glacial refugia (Figure 2). These regions were believed to have provided relatively stable climatic conditions for the *in situ* survival of local plants, such as *Eurycorymbus cavaleriei* (Wang et al., 2009), *J. cathayensis* (Bai et al., 2014), and *C. paliurus* (Kou et al., 2016). Furthermore, Petit et al. (2003) pointed out that populations in refugia not only have relatively high genetic diversity but also contain some unique haplotypes. In our study, it is true for the wild populations MEI, ZHA, and XDS, supporting that the Qinling Mountains, the Wuling Mountains, and the Wu Mountains may have been refugia in western China (e.g., Gong et al., 2008; Wang et al., 2009; Deng et al., 2019).

Our migration vector analyses indicate that *T. vernicifluum* may have experienced a gradual range expansion since the LIG (Figure 5). The northward colonization would provide opportunities for the widespread of a few ancestral haplotypes (H1 and H3) in western and eastern China (Tian et al., 2015). Numerous mountain ranges, especially those near the border between the middle and low “step”, are predicted to be dispersal corridors that have increased the landscape connectivity among populations (Guan et al., 2016). Furthermore, we found that the ESC land bridge may have also acted as a dispersal corridor during the LGM (Figure 2D), as supported by the evidence that Japanese samples share an ancestral haplotype (H3) with those from eastern China (Figure 1A). Such a finding is consistent with that of previous studies showing that there is a close genetic relationship between populations from eastern China and Japan (e.g., *Quercus variabilis*, Chen et al., 2012; *K. septemlobus*, Sakaguchi et al., 2012; *Machilus thunbergii*, Jiang et al., 2021). However, this pattern does not occur in all cases because the submergence of the ECS land bridge may also lead to the divergence of the lineage between eastern China and Japan (e.g., *Ligularia hodgsonii*, Wang et al., 2013; *Platycrater arguta*, Qi et al., 2014; *Euptelea* spp., Cao et al., 2016).

It should be noted that the origin of *T. vernicifluum* in Japan is still controversial (Suzuki et al., 2014). As an economically important arbor species, the lacquer tree was used for natural lacquer collection in China 8,000 years ago (Wu et al., 2018). In Japan, the oldest lacquer was found in Hokkaido 9,000 years ago. Although evidence from fossil woods supported that *T. vernicifluum* grew in middle to northern Honshu of Japan since the Early Jomon Period, it is believed that the species was introduced from China in an earlier time because it does not grow in natural forests and is only found around human settlements (Noshiro and Suzuki, 2004; Noshiro et al., 2007). However, this view is challenged by a recent finding of a fossil wood of *T. vernicifluum* dated back to the incipient Jomon period (~12,600 years ago), from the Torihama shell midden of Fukui prefecture, Japan (Suzuki et al., 2014). This fossil wood did not show evidence of artificial processing, suggesting that *T. vernicifluum* is likely to be native to Japan during the glacial periods (Suzuki et al., 2014). If it is true, we infer that the species may have an origin from eastern China because they share an ancestral haplotype with those from eastern China, and the genetic diversity in Japan is much lower than that in eastern China. Furthermore, it should be noted that our study provides evidence for the historical transplanting of lacquer trees between eastern and western China (Figure 1A). This finding reminds us it is necessary to use both provenance trials and genomic analyses to assess the adaptive ability of lacquer trees to different climatic conditions, which will offer guidance for the future management of *T. vernicifluum* resources.

## Conclusion

*Toxicodendron vernicifluum* exhibits a clear east-west phylogeographic break associated with the stepped geomorphology of China. This break was much sharper than previously reported because no shared haplotypes were detected among the wild trees on the two sides. Furthermore, this break occurred at a much larger scale, extending from subtropical (e.g., the Xuefeng Mountain) to warm-temperate areas (e.g., the Taihang Mountain) of China. Our study supports that the eastern and western clades may have diverged during the Early Pleistocene, suggesting a likely scenario of allopatric divergence in response to long-term geographic isolation across at least two glacial refugia. Combining the evidence from fossil records and molecular analyses, we support that *T. vernicifluum* in Japan may have an origin from eastern China, while the East China Sea may have acted as a dispersal corridor during the glacial periods.

## Data Availability Statement

The datasets presented in this study can be found in online repositories. Sequence data are available on GenBank (http://www.ncbi.nlm.nih.gov/genbank/) under the accession numbers OL355137–OL355141.

## Author Contributions

LW, YF, and FZ conceived and designed this research. LW, YL, SN, MS, TA, KK, LX, and MZ collected samples. LW performed experiments and wrote the original draft. LW, YL, and NH analyzed the data. YF and FZ supervised the project. All authors contributed to the article and approved the submitted version.

## Funding

This research was supported by the National Key Research and Development Program of China (2017YFD060130501), the China Postdoctoral Science Foundation (2020M681629), the Jiangsu Postdoctoral Research Foundation (2021K038A), Shaanxi Innovation Capability Support Plan “Chinese Lacquer Tree Resource Data Information Sharing Platform” (2022PT-05), and the Priority Academic Program Development of Jiangsu Higher Education Institutions (PAPD). LW was supported by the fund from the China Scholarship Council (202008320479).

## Conflict of Interest

The authors declare that the research was conducted in the absence of any commercial or financial relationships that could be construed as a potential conflict of interest.

## Publisher’s Note

All claims expressed in this article are solely those of the authors and do not necessarily represent those of their affiliated organizations, or those of the publisher, the editors and the reviewers. Any product that may be evaluated in this article, or claim that may be made by its manufacturer, is not guaranteed or endorsed by the publisher.

## Footnotes

^1^ https://www.2bulu.com/

^2^ http://www.gbif.org/

^3^ http://www.cvh.ac.cn/

^4^ http://ppbc.iplant.cn/

^5^ https://api.map.baidu.com/lbsapi/getpoint/index.html

^6^ http://worldclim.org

## References

[ref1] Arbogast B. S. Kenagy G. J. (2001). Comparative phylogeography as an integrative approach to historical biogeography. J. Biogeogr. 28, 819–825. doi: 10.1046/j.1365-2699.2001.00594.x

[ref2] Avise J. C. (1992). Molecular population structure and the biogeographic history of a regional fauna: a case history with lessons for conservation biology. Oikos 63, 62–76. doi: 10.2307/3545516

[ref3] Avise J. C. (2000). Phylogeography: The History and Formation of Species. Cambridge: Harvard University Press.

[ref4] Bai G. Q. Li W. M. Chen H. Li B. Li S. F. (2017). Characteristics of molecular evolution of *Toxicodendron vernicifluum* in Qinba Mountains by nrDNA ITS and cpDNA sequence. Bull. Bot. Res. 37, 579–586. doi: 10.7525/j.issn.1673-5102.2017.04.014

[ref5] Bai W. N. Wang W. T. Zhang D. Y. (2014). Contrasts between the phylogeographic patterns of chloroplast and nuclear DNA highlight a role for pollen-mediated gene flow in preventing population divergence in an east Asian temperate tree. Mol. Phylogenet. Evol. 81, 37–48. doi: 10.1016/j.ympev.2014.08.024, PMID: 25196588

[ref6] Bai W. N. Wang W. T. Zhang D. Y. (2016). Phylogeographic breaks within Asian butternuts indicate the existence of a phytogeographic divide in East Asia. New Phytol. 209, 1757–1772. doi: 10.1111/nph.13711, PMID: 26499508

[ref7] Bouckaert R. Vaughan T. G. Barido-Sottani J. Duchêne S. Fourment M. Gavryushkina A.. (2019). BEAST 2.5: an advanced software platform for Bayesian evolutionary analysis. PLoS Comput. Biol. 15:e1006650. doi: 10.1371/journal.pcbi.1006650, PMID: 30958812 PMC6472827

[ref8] Brown J. L. (2014). SDMtoolbox: a python-based GIS toolkit for landscape genetic, biogeographic and species distribution model analyses. Methods Ecol. Evol. 5, 694–700. doi: 10.1111/2041-210X.12200 PMC5721907 29230356

[ref9] Bui T. T. Vu D. D. Dang Q. H. Zhang Y. Huang X. H. (2017). High genetic diversity and population structure of lacquer cultivars (*Toxicodendron vernicifluum*) in Shaanxi province, China revealed by SSR markers. Res. J. Biotechnol. 12, 14–23.

[ref10] Buschbom J. Yanbaev Y. Degen B. (2011). Efficient long-distance gene flow into an isolated relict oak stand. J. Hered. 102, 464–472. doi: 10.1093/jhered/esr023, PMID: 21525180

[ref11] Cab-Sulub L. Álvarez-Castañeda S. T. (2021). Climatic dissimilarity associated with phylogenetic breaks. J. Mammal. 102, 1592–1604. doi: 10.1093/jmammal/gyab108

[ref12] Cao Y. N. Comes H. P. Sakaguchi S. Chen L. Y. Qiu Y. X. (2016). Evolution of East Asia’s Arcto-tertiary relict *Euptelea* (Eupteleaceae) shaped by late Neogene vicariance and quaternary climate change. BMC Evol. Biol. 16:66. doi: 10.1186/s12862-016-0636-x, PMID: 27001058 PMC4802896

[ref13] Chan L. M. Brown J. L. Yoder A. D. (2011). Integrating statistical genetic and geospatial methods brings new power to phylogeography. Mol. Phylogenet. Evol. 59, 523–537. doi: 10.1016/j.ympev.2011.01.020, PMID: 21352934

[ref14] Chen D. Zhang X. Kang H. Sun X. Yin S. Du H.. (2012). Phylogeography of *Quercus variabilis* based on chloroplast DNA sequence in East Asia: multiple glacial refugia and mainland-migrated island populations. PLoS One 7:e47268. doi: 10.1371/journal.pone.0047268, PMID: 23115642 PMC3480369

[ref15] Deng T. Abbott R. J. Li W. Sun H. Volis S. (2019). Genetic diversity hotspots and refugia identified by mapping multi-plant species haplotype diversity in China. Israel J. Plant Sci. 66, 136–151. doi: 10.1163/22238980-20191083

[ref17] Du F. K. Peng X. L. Liu J. Q. Lascoux M. Hu F. S. Petit R. J. (2011). Direction and extent of organelle DNA introgression between two spruce species in the Qinghai-Tibetan plateau. New Phytol. 192, 1024–1033. doi: 10.1111/j.1469-8137.2011.03853.x, PMID: 21883235

[ref18] Dupanloup I. Schneider S. Excoffier L. (2002). A simulated annealing approach to define the genetic structure of populations. Mol. Ecol. 11, 2571–2581. doi: 10.1046/j.1365-294X.2002.01650.x, PMID: 12453240

[ref19] Excoffier L. Laval G. Schneider S. (2005). Arlequin (version 3.0): an integrated software package for population genetics data analysis. Evol. Bioinforma. 1, 47–50. doi: 10.1177/117693430500100003 PMC2658868 19325852

[ref20] Fan D. Hu W. Li B. O. Morris A. B. Zheng M. Soltis D. E.. (2016). Idiosyncratic responses of evergreen broad-leaved forest constituents in China to the late quaternary climate changes. Sci. Rep. 6:31044. doi: 10.1038/srep31044, PMID: 27534981 PMC4989166

[ref22] Fan D. M. Yue J. P. Nie Z. L. Li Z. M. Comes H. P. Sun H. (2013). Phylogeography of *Sophora davidii* (Leguminosae) across the ‘Tanaka-Kaiyong line’, an important phytogeographic boundary in Southwest China. Mol. Ecol. 22, 4270–4288. doi: 10.1111/mec.12388, PMID: 23927411

[ref23] Feliner G. N. (2014). Patterns and processes in plant phylogeography in the Mediterranean Basin: A review. Perspect. Plant Ecol. 16, 265–278. doi: 10.1016/j.ppees.2014.07.002

[ref24] Feng G. Mao L. Sandel B. Swenson N. G. Svenning J. C. (2016). High plant endemism in China is partially linked to reduced glacial-interglacial climate change. J. Biogeogr. 43, 145–154. doi: 10.1111/jbi.12613

[ref25] Gao J. Liu Z. L. Zhao W. Tomlinson K. W. Xia S. W. Zeng Q. Y.. (2021). Combined genotype and phenotype analyses reveal patterns of genomic adaptation to local environments in the subtropical oak *Quercus acutissima*. J. Syst. Evol. 59, 541–556. doi: 10.1111/jse.12568

[ref26] Gao L. M. Möller M. Zhang X. M. Hollingsworth M. L. Liu J. Mill R. R.. (2007). High variation and strong phylogeographic pattern among cpDNA haplotypes in *Taxus wallichiana* (Taxaceae) in China and North Vietnam. Mol. Ecol. 16, 4684–4698. doi: 10.1111/j.1365-294X.2007.03537.x, PMID: 17908214

[ref27] Geffen E. L. I. Anderson M. J. Wayne R. K. (2004). Climate and habitat barriers to dispersal in the highly mobile grey wolf. Mol. Ecol. 13, 2481–2490. doi: 10.1111/j.1365-294X.2004.02244.x, PMID: 15245420

[ref28] Gong W. Chen C. Dobeš C. Fu C. X. Koch M. A. (2008). Phylogeography of a living fossil: Pleistocene glaciations forced *Ginkgo biloba* L.(Ginkgoaceae) into two refuge areas in China with limited subsequent postglacial expansion. Mol. Phylogenet. Evol. 48, 1094–1105. doi: 10.1016/j.ympev.2008.05.003, PMID: 18554931

[ref29] Graves T. Chandler R. B. Royle J. A. Beier P. Kendall K. C. (2014). Estimating landscape resistance to dispersal. Landsc. Ecol. 29, 1201–1211. doi: 10.1007/s10980-014-0056-5

[ref30] Guan B. C. Chen W. Gong X. Wu T. Cai Q. Y. Liu Y. Z.. (2016). Landscape connectivity of *Cercidiphyllum japonicum*, an endangered species and its implications for conservation. Ecol. Inform. 33, 51–56. doi: 10.1016/j.ecoinf.2016.04.002

[ref31] Gugger P. F. Ikegami M. Sork V. L. (2013). Influence of late quaternary climate change on present patterns of genetic variation in valley oak, *Quercus lobata* Née. Mol. Ecol. 22, 3598–3612. doi: 10.1111/mec.12317, PMID: 23802553

[ref32] Guo J. H. Liu S. J. Wu Y. H. Guo Y. K. Wang Z. Wang Y. L.. (2019). Genetic structure of *Toxicodendron vernicifluum* in Southern Shanxi Province based on SSR marker. Mol. Plant Breed. 17, 2950–2955. doi: 10.13271/j.mpb.017.002950

[ref33] Guo Z. T. Sun B. Zhang Z. S. Peng S. Z. Xiao G. Q. Ge J. Y.. (2008). A major reorganization of Asian climate by the early Miocene. Clim. Past 4, 153–174. doi: 10.5194/cp-4-153-2008

[ref34] Guo X. D. Wang H. F. Bao L. Wang T. M. Bai W. N. Ye J. W.. (2014). Evolutionary history of a widespread tree species A cer mono in East Asia. Ecol. Evol. 4, 4332–4345. doi: 10.1002/ece3.1278, PMID: 25540694 PMC4267871

[ref35] Hall T. A. (1999). BioEdit: a user-friendly biological sequence alignment editor and analysis program for windows 95/98/NT. Nucleic Acids Symp. Ser. 41, 95–98.

[ref36] Hashida K. Tabata M. Kuroda K. Otsuka Y. Kubo S. Makino R.. (2014). Phenolic extractives in the trunk of *Toxicodendron vernicifluum*: chemical characteristics, contents and radial distribution. J. Wood Sci. 60, 160–168. doi: 10.1007/s10086-013-1385-8

[ref37] He N. Wang L. Li Y. Fang Y. Zhang F. (2020). The complete chloroplast genome sequence of *Toxicodendron sylvestre* (Anacardiaceae). Mitochondrial DNA B Resour. 5, 2008–2009. doi: 10.1080/23802359.2020.1756960

[ref38] Hewitt G. M. (2004). Genetic consequences of climatic oscillations in the Quaternary. Philos. Trans. R. Soc. Lond. B Bio. Sci. 359, 183–195. doi: 10.1098/rstb.2003.1388, PMID: 15101575 PMC1693318

[ref39] Hickerson M. J. Carstens B. C. Cavender-bares J. Crandall K. A. Graham C. H. Johnson J. B.. (2010). Phylogeography’s past, present, and future: 10 years after Avise, 2000. Mol. Phylogenet. Evol. 54, 291–301. doi: 10.1016/j.ympev.2009.09.016, PMID: 19755165

[ref40] Hijmans R. J. (2019). Introduction to the ‘raster’ package (version 2.8-19).

[ref42] Hu L. J. Uchiyama K. Shen H. L. Saito Y. Tsuda Y. Ide Y. (2008). Nuclear DNA microsatellites reveal genetic variation but a lack of phylogeographical structure in an endangered species, *Fraxinus mandshurica*, across north-East China. Ann. Bot. 102, 195–205. doi: 10.1093/aob/mcn074, PMID: 18477559 PMC2712365

[ref43] Jaramillo-Correa J. P. Beaulieu J. Khasa D. P. Bousquet J. (2009). Inferring the past from the present phylogeographic structure of north American forest trees: seeing the forest for the genes. Can. J. For. Res. 39, 286–307. doi: 10.1139/X08-181

[ref44] Jiang K. Tong X. Ding Y. Q. Wang Z. W. Miao L. Y. Xiao Y. E.. (2021). Shifting roles of the East China Sea in the phylogeography of red nanmu in East Asia. J. Biogeogr. 48, 2486–2501. doi: 10.1111/jbi.14215

[ref45] Jiang F. Wu X. (1993). Fundamental characteristics of the stepped landform in China continent. Mar. Geol. Quat. Geol. 13, 15–24.

[ref46] Jiménez-Valverde A. Lobo J. M. (2007). Threshold criteria for conversion of probability of species presence to either–or presence–absence. Acta Oecol. 31, 361–369. doi: 10.1016/j.actao.2007.02.001

[ref47] Kalyaanamoorthy S. Minh B. Q. Wong T. K. Von Haeseler A. Jermiin L. S. (2017). ModelFinder: fast model selection for accurate phylogenetic estimates. Nat. Methods 14, 587–589. doi: 10.1038/nmeth.4285, PMID: 28481363 PMC5453245

[ref48] Kim K. H. Moon E. Choi S. U. Pang C. Kim S. Y. Lee K. R. (2015). Identification of cytotoxic and anti-inflammatory constituents from the bark of *Toxicodendron vernicifluum* (stokes) F. A. Barkley. J. Ethnopharmacol. 162, 231–237. doi: 10.1016/j.jep.2014.12.071, PMID: 25582488

[ref49] Kou Y. Cheng S. Tian S. Li B. Fan D. Chen Y.. (2016). The antiquity of *Cyclocarya paliurus* (Juglandaceae) provides new insights into the evolution of relict plants in subtropical China since the late early Miocene. J. Biogeogr. 43, 351–360. doi: 10.1111/jbi.12635

[ref50] Leigh J. W. Bryant D. (2015). POPART: full-feature software for haplotype network construction. Methods Ecol. Evol. 6, 1110–1116. doi: 10.1111/2041-210X.12410

[ref51] Li Y. Tang Y. Wu T. (2020). The complete chloroplast genome of *Toxicodendron griffithii*. Mitochondrial DNA B Resour. 5, 2211–2212. doi: 10.1080/23802359.2020.1768931, PMID: 33366976 PMC7510646

[ref52] Li Y. Zhang X. Fang Y. (2019). Landscape features and climatic forces shape the genetic structure and evolutionary history of an oak species (*Quercus chenii*) in East China. Front. Plant Sci. 10:1060. doi: 10.3389/fpls.2019.01060, PMID: 31552065 PMC6734190

[ref53] Li M. C. Zhang Y. Q. Meng C. W. Gao J. G. Xie C. J. Liu J. Y.. (2021). Traditional uses, phytochemistry, and pharmacology of *Toxicodendron vernicifluum* (stokes) F. A. Barkley-a review. J. Ethnopharmacol. 267:113476. doi: 10.1016/j.jep.2020.113476, PMID: 33075438

[ref54] Li Y. Zhang X. Wang L. Sork V. L. Mao L. Fang Y. (2022). Influence of Pliocene and Pleistocene climates on hybridization patterns between two closely related oak species in China. Ann. Bot. 129, 231–245. doi: 10.1093/aob/mcab140, PMID: 34893791 PMC8796672

[ref55] Li J. Zhao M. Wei S. Luo Z. Wu H. (2015). Geologic events coupled with Pleistocene climatic oscillations drove genetic variation of Omei treefrog (*Rhacophorus omeimontis*) in southern China. BMC Evol. Biol. 15:289. doi: 10.1186/s12862-015-0572-1, PMID: 26690899 PMC4687352

[ref56] Liao Y. Y. Guo Y. H. Chen J. M. Wang Q. F. (2014). Phylogeography of the widespread plant *Ailanthus altissima* (Simaroubaceae) in China indicated by three chloroplast DNA regions. J. Syst. Evol. 52, 175–185. doi: 10.1111/jse.12065

[ref57] Librado P. Rozas J. (2009). DnaSP v5: a software for comprehensive analysis of DNA polymorphism data. Bioinformatics 25, 1451–1452. doi: 10.1093/bioinformatics/btp187, PMID: 19346325

[ref58] Liu C. Tsuda Y. Shen H. Hu L. Saito Y. Ide Y. (2014). Genetic structure and hierarchical population divergence history of *Acer mono* var. *mono* in south and Northeast China. PLoS One 9:e87187. doi: 10.1007/s00468-011-0581-7, PMID: 24498039 PMC3909053

[ref59] Luo S. He Y. Ning G. Zhang J. Ma G. Bao M. (2011). Genetic diversity and genetic structure of different populations of the endangered species *Davidia involucrata* in China detected by inter-simple sequence repeat analysis. Trees 25, 1063–1071. doi: 10.1007/s00468-011-0581-7

[ref60] Luo D. Xu B. Li Z. M. Sun H. (2021). Biogeographical divides delineated by the three-step landforms of China and the East China Sea: insights from the phylogeography of *Kerria japonica*. J. Biogeogr. 48, 372–385. doi: 10.1111/jbi.14002

[ref61] Luo D. Yue J. P. Sun W. G. Xu B. Li Z. M. Comes H. P.. (2016). Evolutionary history of the subnival flora of the Himalaya-Hengduan Mountains: first insights from comparative phylogeography of four perennial herbs. J. Biogeogr. 43, 31–43. doi: 10.1111/jbi.12610

[ref62] McCauley D. E. (1994). Contrasting the distribution of chloroplast DNA and allozyme polymorphism among local populations of *Silene alba*: implications for studies of gene flow in plants. Proc. Natl. Acad. Sci. U. S. A. 91, 8127–8131. doi: 10.1073/pnas.91.17.8127, PMID: 11607493 PMC44558

[ref63] Noshiro S. Suzuki M. (2004). *Rhus verniciflua* stokes grew in Japan since the early Jomon period. Jpn. J. Hist. Bot. 12, 3–11. doi: 10.34596/hisbot.12.1_3

[ref64] Noshiro S. Suzuki M. Sasaki Y. (2007). Importance of *Rhus verniciflua* Stokes (lacquer tree) in prehistoric periods in Japan, deduced from identification of its fossil woods. Veg. Hist. Archaeobotany 16, 405–411. doi: 10.1007/s00334-006-0058-6

[ref65] Peakall R. Smouse P. E. (2012). GenAlEx 6.5: genetic analysis in excel. Population genetic software for teaching and research-an update. Bioinformatics 28, 2537–2539. doi: 10.1111/j.1471-8286.2005.01155.x, PMID: 22820204 PMC3463245

[ref66] Petit R. J. Aguinagalde I. de Beaulieu J. L. Bittkau C. Brewer S. Cheddadi R.. (2003). Glacial refugia: hotspots but not melting pots of genetic diversity. Science 300, 1563–1565. doi: 10.1126/science.1083264 12791991

[ref67] Phillips S. J. Dudík M. Schapire R. E. (2018). Maxent software for modeling species niches and distributions. Available at: http://biodiversityinformatics.amnh.org/open_source/maxent/ (Accessed June 15, 2021).

[ref68] Pons O. Petit R. J. (1996). Measuring and testing genetic differentiation with ordered versus unordered alleles. Genetics 144, 1237–1245. doi: 10.1093/genetics/144.3.1237, PMID: 8913764 PMC1207615

[ref69] Posada D. Crandall K. A. (2001). Intraspecific gene genealogies: trees grafting into networks. Trends Ecol. Evol. 16, 37–45. doi: 10.1016/S0169-5347(00)02026-7, PMID: 11146143

[ref70] Qi X. S. Yuan N. Comes H. P. Sakaguchi S. Qiu Y. X. (2014). A strong ‘filter’ effect of the East China Sea land bridge for East Asia’s temperate plant species: inferences from molecular phylogeography and ecological niche modelling of *Platycrater arguta* (Hydrangeaceae). BMC Evol. Biol. 14:41. doi: 10.1186/1471-2148-14-41, PMID: 24593236 PMC4015774

[ref71] Qian H. Ricklefs R. E. (2000). Large-scale processes and the Asian bias in species diversity of temperate plants. Nature 407, 180–182. doi: 10.1038/35025052, PMID: 11001054

[ref72] Qiu Y. X. Fu C. X. Comes H. P. (2011). Plant molecular phylogeography in China and adjacent regions: tracing the genetic imprints of quaternary climate and environmental change in the world’s most diverse temperate flora. Mol. Phylogenet. Evol. 59, 225–244. doi: 10.1016/j.ympev.2011.01.012, PMID: 21292014

[ref73] Qiu Y. X. Lu Q. X. Zhang Y. H. Cao Y. N. (2017). Phylogeography of East Asia’s tertiary relict plants: current progress and future prospects. Biodivers. Sci. 25, 24–28. doi: 10.17520/biods.2016292

[ref74] R Core Team (2018). R: A language and environment for statistical computing.

[ref75] Rambaut A. Drummond A. J. Xie D. Baele G. Suchard M. A. (2018). Posterior summarisation in Bayesian phylogenetics using tracer 1.7. Syst. Biol. 67, 901–904. doi: 10.1093/sysbio/syy032, PMID: 29718447 PMC6101584

[ref76] Rendell S. Ennos R. A. (2003). Chloroplast DNA diversity of the dioecious European tree *Ilex aquifolium* L. (English holly). Mol. Ecol. 12, 2681–2688. doi: 10.1046/j.1365-294X.2003.01934.x, PMID: 12969471

[ref77] Sakaguchi S. Qiu Y. X. Liu Y. H. Qi X. S. Kim S. H. Han J.. (2012). Climate oscillation during the quaternary associated with landscape heterogeneity promoted allopatric lineage divergence of a temperate tree *Kalopanax septemlobus* (Araliaceae) in East Asia. Mol. Ecol. 21, 3823–3838. doi: 10.1111/j.1365-294X.2012.05652.x, PMID: 22646502

[ref78] Schaal B. A. Hayworth D. A. Olsen K. M. Rauscher J. T. Smith W. A. (1998). Phylogeographic studies in plants: problems and prospects. Mol. Ecol. 7, 465–474. doi: 10.1046/j.1365-294x.1998.00318.x

[ref79] Schoener T. W. (1968). The Anolis lizards of Bimini: resource partitioning in a complex fauna. Ecology 49, 704–726. doi: 10.2307/1935534

[ref80] Shafer A. B. Cullingham C. I. Cote S. D. Coltman D. W. (2010). Of glaciers and refugia: a decade of study sheds new light on the phylogeography of northwestern North America. Mol. Ecol. 19, 4589–4621. doi: 10.1111/j.1365-294X.2010.04828.x, PMID: 20849561

[ref81] Shi M. M. Michalski S. G. Welk E. Chen X. Y. Durka W. (2014). Phylogeography of a widespread Asian subtropical tree: genetic east–west differentiation and climate envelope modelling suggest multiple glacial refugia. J. Biogeogr. 41, 1710–1720. doi: 10.1111/jbi.12322

[ref82] Soltis D. E. Gitzendanner M. A. Strenge D. D. Soltis P. S. (1997). Chloroplast DNA intraspecific phylogeography of plants from the Pacific northwest of North America. Plant Syst. Evol. 206, 353–373. doi: 10.1007/BF00987957

[ref83] Soltis D. E. Morris A. B. McLachlan J. S. Manos P. S. Soltis P. S. (2006). Comparative phylogeography of unglaciated eastern North America. Mol. Ecol. 15, 4261–4293. doi: 10.1111/j.1365-294X.2006.03061.x, PMID: 17107465

[ref84] Sun Y. B. (2017). FasParser: a package for manipulating sequence data. Zool. Res. 38, 110–112. doi: 10.24272/j.issn.2095-8137.2017.017, PMID: 28409507 PMC5396028

[ref85] Sun Y. Hu H. Huang H. Vargas-Mendoza C. F. (2014). Chloroplast diversity and population differentiation of *Castanopsis fargesii* (Fagaceae): a dominant tree species in evergreen broad-leaved forest of subtropical China. Tree Genet. Genomes 10, 1531–1539. doi: 10.1007/s11295-014-0776-3

[ref86] Suzuki M. Noshiro S. Tanaka T. Kobayashi K. Wang Y. Liu J. Q.. (2014). Origin of Urushi (*Toxicodendron vernicifluum*) in the Neolithic Jomon period of Japan. Bull. Natl. Mus. Jpn. Hist. 187, 49–70. doi: 10.34596/hisbot.15.1_58

[ref87] Suzuki M. Yonekura K. Noshiro S. (2007). Distribution and habitat of *Toxicodendron vernicifluum* (stokes) F. A. Barkl. (Anacardiaceae) in China. Jpn. J. Hist. Bot. 15, 58–62. doi: 10.34596/hisbot.15.1_58

[ref88] Taberlet P. Fumagalli L. Wust-Saucy A. G. Cosson J. F. (1998). Comparative phylogeography and postglacial colonization routes in Europe. Mol. Ecol. 7, 453–464. doi: 10.1046/j.1365-294x.1998.00289.x, PMID: 9628000

[ref89] Tian S. Lei S. Q. Hu W. Deng L. L. Li B. O. Meng Q. L.. (2015). Repeated range expansions and inter-/postglacial recolonization routes of Sargentodoxa cuneata (Oliv.) Rehd. et Wils.(Lardizabalaceae) in subtropical China revealed by chloroplast phylogeography. Mol. Phylogenet. Evol. 85, 238–246. doi: 10.1016/j.ympev.2015.02.016, PMID: 25732070

[ref90] Vu D. D. Bui T. T. Nguyen T. H. (2018). Isolation and characterization of polymorphic microsatellite markers in *Toxicodendron vernicifluum*. Czech J. Genet. Plant Breed. 54, 17–25. doi: 10.17221/183/2016-CJGPB

[ref91] Walker S. Williams J. Lear J. Beck M. (2008). FS11.5 *Toxicodendron* dermatitis in the United Kingdom. Contact Dermatitis 50:163. doi: 10.1111/j.0105-1873.2004.0309cy.x

[ref92] Wan T. F. (2012). The Tectonics of China: Data, Maps and Evolution. Berlin: Springer Science & Business Media.

[ref93] Wang J. Gao P. Kang M. Lowe A. J. Huang H. (2009). Refugia within refugia: the case study of a canopy tree (*Eurycorymbus cavaleriei*) in subtropical China. J. Biogeogr. 36, 2156–2164. doi: 10.1111/j.1365-2699.2009.02165.x

[ref94] Wang J. F. Gong X. Chiang Y. C. Kuroda C. (2013). Phylogenetic patterns and disjunct distribution in *Ligularia hodgsonii* hook. (Asteraceae). J. Biogeogr. 40, 1741–1754. doi: 10.1111/jbi.12114

[ref95] Wang L. He N. Li Y. Fang Y. M. Zhang F. L. (2020a). Complete chloroplast genome sequence of Chinese lacquer tree (*Toxicodendron vernicifluum*, Anacardiaceae) and its phylogenetic significance. Biomed. Res. Int. 2020:9014873. doi: 10.1155/2020/9014873, PMID: 32071921 PMC7011389

[ref96] Wang L. He N. Li Y. Fang Y. M. Zhang F. L. (2020b). The complete chloroplast genome sequence of *Toxicodendron succedaneum* (Anacardiaceae). Mitochondrial DNA B Resour. 5, 1956–1957. doi: 10.1080/23802359.2020.1756956

[ref97] Wang Y. H. Jiang W. M. Comes H. P. Hu F. S. Qiu Y. X. Fu C. X. (2015a). Molecular phylogeography and ecological niche modelling of a widespread herbaceous climber, *Tetrastigma hemsleyanum* (Vitaceae): insights into Plio–Pleistocene range dynamics of evergreen forest in subtropical China. New Phytol. 206, 852–867. doi: 10.1111/nph.13261 25639152

[ref98] Wang Y. Liu K. Bi D. Zhou S. Shao J. (2018). Molecular phylogeography of east Asian *Boea clarkeana* (Gesneriaceae) in relation to habitat restriction. PLoS One 13:e0199780. doi: 10.1371/journal.pone.0199780, PMID: 29969490 PMC6029794

[ref99] Wang W. Tian C. Y. Li Y. H. Li Y. (2015b). Molecular data and ecological niche modelling reveal the phylogeographic pattern of *Cotinus coggygria* (Anacardiaceae) in china's warm-temperate zone. Plant Biol. 16, 1114–1120. doi: 10.1111/plb.12157, PMID: 24494998

[ref100] Warren D. L. Glor R. E. Turelli M. (2008). Environmental niche equivalency versus conservatism: quantitative approaches to niche evolution. Evolution 62, 2868–2883. doi: 10.1111/j.1558-5646.2008.00482.x, PMID: 18752605

[ref101] Warren D. L. Glor R. E. Turelli M. (2010). ENMTools: a toolbox for comparative studies of environmental niche models. Ecography 33, 607–611. doi: 10.1111/j.1600-0587.2009.06142.x

[ref102] Watanabe A. Tamura M. Izumi Y. Yamaguchi R. Iki T. Tabata M. (2019). Evaluation of genetic diversity of *Toxicodendron vernicifluum* planted in Japan using EST-SSR and genetic SSR markers. J. Jpn For. Soc. 101, 298–304. doi: 10.4005/jjfs.101.298

[ref103] Wei S. Zhao X. Tian M. Li L. Hu Z. (2010). Application of plant morphology and AFLP molecular markers to identify *Toxicodendron vernicifluum* varieties of Shaanxi. Xi Bei Zhi Wu Xue Bao 30, 665–671.

[ref104] Wolfe K. H. Li W. H. Sharp P. M. (1987). Rates of nucleotide substitution vary greatly among plant mitochondrial, chloroplast, and nuclear DNAs. Proc. Natl. Acad. Sci. U. S. A. 84, 9054–9058. doi: 10.1073/pnas.84.24.9054, PMID: 3480529 PMC299690

[ref105] Wu M. Zhang B. Jiang L. Wu J. Sun G. (2018). Natural lacquer was used as a coating and an adhesive 8000 years ago, by early humans at Kuahuqiao, determined by ELISA. J. Archaeol. Sci. 100, 80–87. doi: 10.1016/j.jas.2018.10.004

[ref106] Yang A. Zhong Y. Liu S. Liu L. Liu T. Li Y.. (2019). New insight into the phylogeographic pattern of *Liriodendron chinense* (Magnoliaceae) revealed by chloroplast DNA: east-west lineage split and genetic mixture within western subtropical China. PeerJ 7:e6355. doi: 10.7717/peerj.6355, PMID: 30723627 PMC6361005

[ref107] Ye J. W. Bai W. N. Bao L. Wang T. M. Wang H. F. Ge J. P. (2017b). Sharp genetic discontinuity in the aridity-sensitive *Lindera obtusiloba* (Lauraceae): solid evidence supporting the tertiary floral subdivision in East Asia. J. Biogeogr. 44, 2082–2095. doi: 10.1111/jbi.13020

[ref108] Ye J. W. Li D. Z. (2021). Distinct late Pleistocene subtropical-tropical divergence revealed by fifteen low-copy nuclear genes in a dominant species in south-East China. Sci. Rep. 11:4147. doi: 10.1038/s41598-021-83473-w, PMID: 33603069 PMC7892551

[ref109] Ye J. W. Zhang Y. Wang X. J. (2017a). Phylogeographic breaks and the mechanisms of their formation in the Sino-Japanese floristic region. Chin. J. Plant Ecol. 41, 1003–1019. doi: 10.17521/cjpe.2016.0388

[ref110] Yin X. Qian H. Sui X. Zhang M. Mao L. Svenning J. C.. (2021). Effects of climate and topography on the diversity anomaly of plants disjunctly distributed in eastern Asia and eastern North America. Glob. Ecol. Biogeogr. 30, 2029–2042. doi: 10.1111/geb.13366

[ref111] Yu H. B. Zhang Y. L. Liu L. S. Qi W. Li S. C. Hu Z. J. (2015). Combining the least cost path method with population genetic data and species distribution models to identify landscape connectivity during the late quaternary in Himalayan hemlock. Ecol. Evol. 5, 5781–5791. doi: 10.1002/ece3.1840, PMID: 26811753 PMC4717335

[ref112] Zeng Y. F. Wang W. T. Liao W. J. Wang H. F. Zhang D. Y. (2015). Multiple glacial refugia for cool-temperate deciduous trees in northern East Asia: The *Mongolian* oak as a case study. Mol. Ecol. 24, 5676–5691. doi: 10.1111/mec.13408, PMID: 26439083

[ref113] Zhang X. W. Li Y. Liu C. Xia T. Zhang Q. Fang Y. M. (2015). Phylogeography of the temperate tree species *Quercus acutissima* in China: inferences from chloroplast DNA variations. Biochem. Syst. Ecol. 63, 190–197. doi: 10.1016/j.bse.2015.10.010

[ref114] Zhang X. W. Li Y. Zhang Q. Fang Y. M. (2018). Ancient east-west divergence, recent admixture, and multiple marginal refugia shape genetic structure of a widespread oak species (*Quercus acutissima*) in China. Tree Genet. Genomes 14:88. doi: 10.1007/s11295-018-1302-9

[ref115] Zhang F. L. Zhang W. Q. Wei S. N. (2007). Research and fine application of lacquer tree resources in China. J. Chin. Lacquer 2, 36–50. doi: 10.19334/j.cnki.issn.1000-7067.2007.02.005

[ref116] Zhao M. Liu C. Zheng G. (2013). Comparative studies of bark structure, lacquer yield and urushiol content of cultivated *Toxicodendron vernicifluum* varieties. N. Z. J. Bot. 51, 13–21. doi: 10.1080/0028825X.2012.731005

[ref117] Zhong Y. Yang A. Liu S. Liu L. Li Y. Wu Z.. (2019). RAD-Seq data point to a distinct split in *Liriodendron* (Magnoliaceae) and obvious east–west genetic divergence in *L. chinense*. Forests 10:13. doi: 10.3390/f10010013

