# Supplementary material for: Stepped Geomorphology Shaped the Phylogeographic Structure of a Widespread Tree Species (Toxicodendron vernicifluum, Anacardiaceae) in East Asia
Source: Front Plant Sci. 2022 Jun 2;13:920054. doi: 10.3389/fpls.2022.920054 (PMC9201781; doi:10.3389/fpls.2022.920054)

**Figure S1 BEAST-derived chronogram for five chloroplast DNA haplotypes of *Toxicodendron vernicifluum*, with *T. radicans* as an outgroup. Ages and their 95% highest posterior density (HPD) credibility intervals and posterior probabilities (PP) are labeled for each node.**


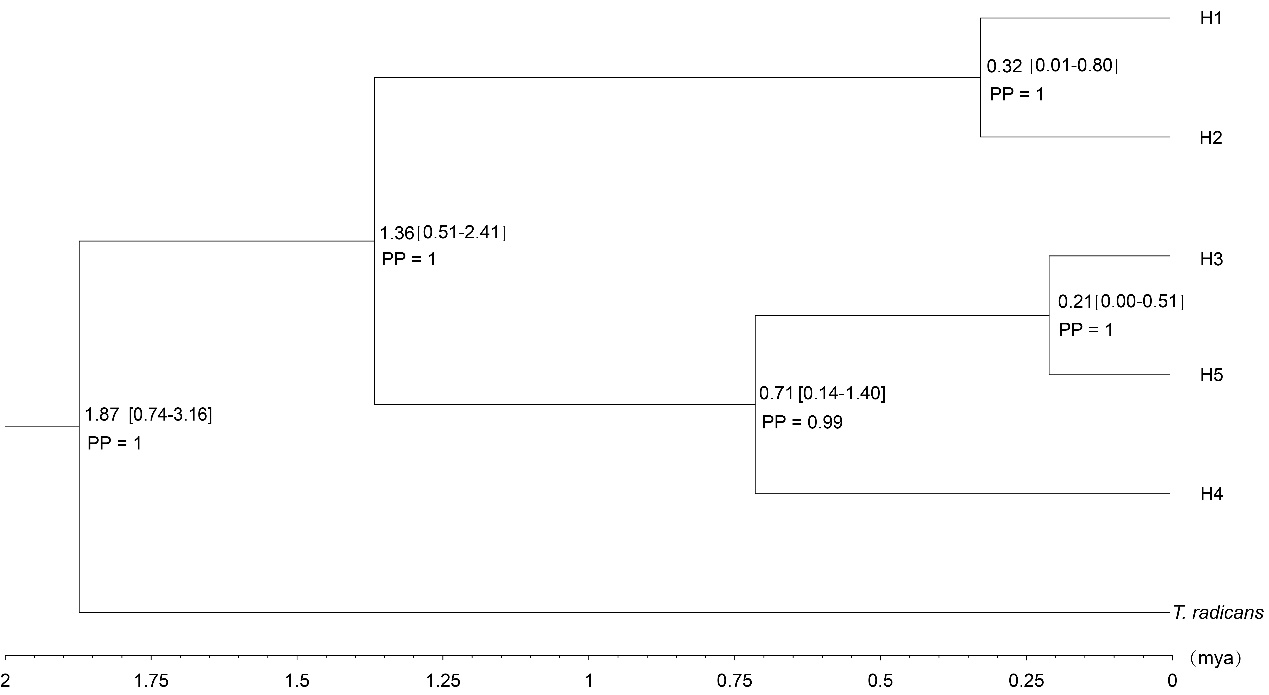

Supplement: Supplementary file 2 [file Data_Sheet_2.docx]
